# Bioactive Phenolic Compounds from the Agroindustrial Waste of Colombian Mango Cultivars ‘Sugar Mango’ and ‘Tommy Atkins’—An Alternative for Their Use and Valorization

**DOI:** 10.3390/antiox8020041

**Published:** 2019-02-15

**Authors:** Henry I. Castro-Vargas, Diego Ballesteros Vivas, Jenny Ortega Barbosa, Sandra Johanna Morantes Medina, Fabio Aristizabal Gutiérrez, Fabián Parada-Alfonso

**Affiliations:** 1 Faculty of Engineering, Universidad Libre, Seccional Bogotá, Carrera 70 No 53-40, Bogotá D.C. 111071, Colombia; 2 Department of Chemistry, Faculty of Sciences, Universidad Nacional de Colombia, Carrera 30 No 45-03, Bogotá D.C. 111321, Colombia; dballesterosv@unal.edu.co (D.B.V.); jportegab@unal.edu.co (J.O.B.); fparadaa@unal.edu.co (F.P.-A.); 3 Unit of Basic Oral Investigation (UIBO), School of Dentistry, Universidad El Bosque, Av. Cra 9 No. 131 A-02, Bogotá D.C. 110121, Colombia; smorantes@unbosque.edu.co; 4 Department of Farmacy, Faculty of Sciences, Universidad Nacional de Colombia, Carrera 30 No 45-03, Bogotá D.C. 111321, Colombia; faaristizabalg@unal.edu.co

**Keywords:** *Mangifera indica* L., agro-food by-products, natural antioxidants, antiproliferative phenolics, mangiferin, galloyl glucosides

## Abstract

The aim of this study was to explore the potential of the agroindustrial waste from two Colombian mango cultivars as sources of bioactive phenolic compounds. Phenolic extracts from mango waste (peels, seed coats, and seed kernels) of ‘sugar mango’ and ‘Tommy Atkins’ cultivars were obtained. The bioactive properties of the phenolic extracts were accessed by measuring their free radical scavenging activity and antioxidant effects against lipid oxidation in food products; moreover, their antiproliferative effects against some cell lines of human cancer were explored. It is observed that the agroindustrial waste studied provides promising sources of bioactive phenolics. ‘Sugar mango’ waste provided extracts with the highest antioxidant effect in food products and antiproliferative activity; these extracts reduced lipid oxidation and cell growth by more than 57% and 75%, respectively. The seed kernel from ‘sugar mango’ supplied the extract with the best bioactive qualities; in addition, some recognized bioactive phenolics (such as mangiferin and several galloyl glucosides) were observed in this extract and related with its properties. The results obtained suggest that ‘sugar mango’ waste may be considered a source of bioactive phenolics, with promising uses in food and pharmaceutical products. Thus, a suitable alternative for the use and valorization of agroindustrial waste from Colombian mango cultivars is presented.

## 1. Introduction

**Figure 1:**
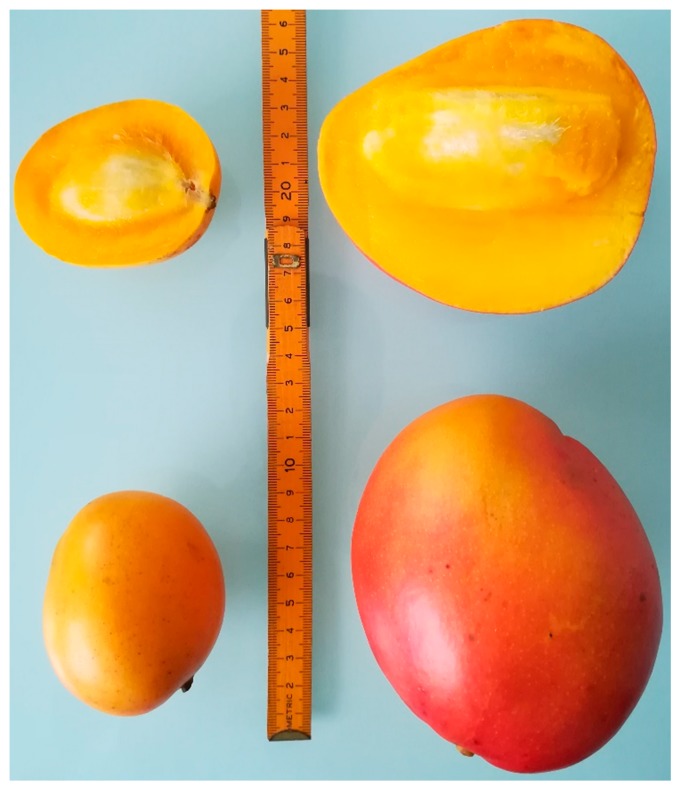
Colombian mango cultivars: ‘sugar mango’ (**left**) and ‘Tommy Atkins’ (**right**).

Mango (*Mangifera indica* L.) is recognized as one of the most important tropical and subtropical fruits in the world. It is produced in large quantities and highly accepted by consumers. Nowadays, the production of mango takes place in more than 115 countries [1]. According to the Food and Agriculture Organization (FAO) of the United Nations, the global production of mango was 46.5 million metric tons in 2016; India was the main mango producer, at 18.7 million metric tons [2]. There are several hundreds of mango cultivars in the world; some of them are endemic to certain regions and countries (e.g., South America or Asia). In Colombia, more than 20 different cultivars have been described [3], of which ‘sugar mango’ and ‘Tommy Atkins’ (see Figure 1) are very important for the Colombian agroindustry. ‘Sugar mango’ is a small Colombian variety that has high demand in the United States and Europe due to its low fiber content and pleasant aroma and taste [4]. ‘Tommy Atkins’ is a mango cultivar that is present in many regions of America and Asia [1,5,6], recognized for its large size and common use in the food industry. Colombia produced about 315,000 metric tons of mango in 2016 [2]; however, its production has increased in the last years in order to supply the industrial and export requirements. Between 35% and 45% of Colombian mango production corresponds to the ‘sugar mango’ and ‘Tommy Atkins’ cultivars; these are principally consumed fresh (including for export) and processed into food products such as nectar, jam, frozen mango, dehydrated products, and canned slices.

The industrial processing of agro-food products produces high amounts of waste. On average, it is estimated that 30–50% of processed food is discarded as waste. The agroindustrial uses of fruits produce large amounts of pomace, peels, seeds, and kernels. Worldwide, fruit processing generates more than 0.6 billion tons of waste/year; the wine and beverage industries are the main waste producers. During the industrial processing of mango, high amounts of peels and seeds are generated. Previous reports cited that the commercial processing of ‘sugar mango’ and ‘Tommy Atkins’ produces approximately 40–60% waste [7]. The mean rate of the generation of mango agroindustrial waste in Colombia is 157,500 tons per year; about 63,000 tons correspond to ‘sugar mango’ and ‘Tommy Atkins’ waste. Mango waste generates environmental pollution; in addition, appropriate disposal involves high costs for agro-industries.

The consumers’ interest in healthy food has increased, and there is a high demand for supplement foods with polyphenols, carotenoids, or tocopherols obtained from natural sources. Agroindustrial waste from processing fruits is well known as a source of high-value compounds with wide variety of bioactive properties and promising uses. Seeds and kernels are rich in mono- or polyunsaturated fatty acids, while pomace, peels, and some seeds are rich in antioxidants [8]. Polyphenols recovered from some agroindustrial waste such as grape pomace [9], cocoa bean hulls [10,11], banana peels [12], and berry pomace [13] have been studied as preservatives and nutraceutical ingredients in food products.

Mango agroindustrial waste contains significant amounts of high-value phytochemicals, which makes it suitable to be processed and used in food and pharmaceutical products. Mango peels are rich in phenolic compounds and carotenoids [1,5,6,14,15,16], whereas mango seed kernels are good sources of polyunsaturated fatty acids and phenolics such as ellagic acid, gallic acid, and its derivates, coumarin and mangiferin [1,5,17,18,19]. Previous studies have shown that phenolics from mango peels and kernels exhibit excellent bioactive properties against some chronic-degenerative diseases related to oxidative stress. Phenolic acids, flavonoids, and gallic acid derivates have anti-inflammatory, antioxidant, and anticancer activities [5,6,14,20,21,22]. Mangiferin is the most recognized phenolic compound from mango waste due to its pharmacological properties including antioxidant, antiproliferative, and antiviral activities [23]. Agroindustrial waste from ‘Tommy Atkins’ mango (Ecuadorian and Indian cultivars) has been explored as sources of phenolic compounds; these studies showed that the extracts from peels and kernels have good antioxidant properties [1,6]. It is worth mentioning that previous reports on possible uses of agroindustrial waste generated from the Colombian mango cultivars ‘sugar mango’ and ‘Tommy Atkins’ were not found. ‘Sugar mango’ is a cultivar originally from Colombia and studies are in development. Hence, to explore the potential of agroindustrial waste from Colombian mango cultivars, the use of ‘sugar mango’ and ‘Tommy Atkins’ as sources of bioactive phenolic compounds is a suitable option. The utilization of mango waste can be an economical means to reduce the problem of waste disposal from mango agro-industries, as well as a way to obtain a functional natural product for the food and pharmaceutical industries. Additionally, the valorization of mango waste would have a positive socio-economical effect on Colombian mango producers, contributing to improve the mango productive chain.

Considering the above, the aim of the present study was to explore the potential of the agroindustrial waste from Colombian mango cultivars as sources of bioactive phenolic compounds with promising uses in food and pharmaceutical products. Extracts rich in phenolics were obtained from mango waste and their total contents of phenolics and flavonoids were evaluated. The bioactive properties of extracts were accessed by measuring their free radical scavenging activity and antioxidant effects against the lipid oxidation in food products; in addition, their antiproliferative effect against some cell lines of human cancer were explored. Finally, the extract with the best qualities was analyzed by high-performance liquid chromatography–electrospray ionization-mass spectrometry (HPLC–ESI-MS) in order to identify some phenolic compounds, and relate these substances with the antioxidant and antiproliferative results.

## 2. Materials and Methods

### 2.1. Mango Waste Samples and Phenolic Extraction

The mango waste samples (peels and seeds) were obtained from the agroindustrial processing of mango fruits (‘sugar mango’ and ‘Tommy Atkins’); these were supplied by the agro-food companies Pulpafruit S.A. and Alimentos SAS S.A. (Bogotá, Colombia). These companies collected the mango fruits in several locations at north and center of Colombia (e.g., the Magdalena, Cundinamarca, and Tolima Departments). In general, the fruits were grown between 500 and 1200 meters above sea level, and temperature ranged from 20 °C to 35 °C. The mango waste samples were randomly sampled for 12 months; for each kind of waste 30 kg was collected, and the samples were cleaned and dried at room temperature (15 °C). The dry seeds were split into coat and kernel (endosperm); then, every dried sample (peel, seed coat, and seed kernel) was ground using a grain mill (Corona-Universal, Bogotá, Colombia). The particles that were obtained were separated using sieves and a vibratory stirrer, and the resulting materials with size between 0.180 and 0.850 mm (Mesh −16/+80 U.S. standard size sieves) were selected to obtain the phenolic extracts. Six different mango waste samples were obtained and used in the phenolic extraction: ‘sugar mango’ peel (PS), ‘sugar mango’ seed coat (SCS), ‘sugar mango’ seed kernel (SKS), ‘Tommy Atkins’ mango peel (PT), ‘Tommy Atkins’ mango seed coat (SCT), and ‘Tommy Atkins’ mango seed kernel (SKT).

The Soxhlet method was used as an exhaustive extraction process of the phenolic compound from mango waste. Methanol was used as solvent to aim at recovering high amounts of phenolic substances; however, for food and pharmaceutical applications other solvents such as ethanol, water, or their mixtures should be used. The extractions were performed under reduced pressure in order to avoid the thermal degradation of the target compounds. The pressure of the extraction system was adjusted at 0.30 atm in order to reduce the methanol boiling point at 35 °C ± 1 °C. Each waste sample (10.0 g) was extracted for 24 hours under continuous solvent reflux. After the extractions, the solvent was removed from the extracts using vacuum; then, the extracts were frozen and lyophilized in a freeze dryer (Labconco Corp., Kansas City, MO, USA). The dry extracts were stored at −20 °C until measurement of their total phenolic content (TPC), total flavonoid content (TFC), antioxidant activity (AA), and antiproliferative activity.

### 2.2. Determination of Total Phenolic Content

The TPC in all the extracts was evaluated using the Folin–Ciocalteu method following the procedure that was described by Hosu et al. [24] with some modifications. All dry extracts were resuspended in ethanol at 20 mg/mL, and 100 μL of each extract solution was mixed with 750 μL of 10% w/w Folin–Ciocalteu reagent solution. After 5 min, 750 μL of 6% w/w sodium carbonate solution was added; the mixture was stirred and left to react in the dark at room temperature for 90 min. After the reaction time, the absorbance at 765 nm was measured (Thermo Scientific Evolution 600 UV/Vis, Madison, WI, USA). Tests were run in triplicate and the results are presented as mg of gallic acid equivalents per 100 g of raw material (mg GAE/100 g).

### 2.3. Determination of Total Flavonoid Content

The TFC in all extracts was determined following the procedure developed by Chang et al. [25]. A 500-μL sample of each ethanolic extract solution (20 mg/mL) was mixed with 1.5 mL of 95% w/w ethanol, 100 μL of 10% w/w aluminium chloride, 100 μL of 1 M sodium acetate, and 2.8 mL of water. The mixture was stirred and incubated at room temperature for 30 min. After the reaction time, the absorbance was measured at 415 nm (Thermo Scientific Evolution 600 UV/Vis). The TFC results are presented as mg of quercetin per 100 g of raw material (mg quer/100 g), calculated using a quercetin standard curve. All measurements were carried out in triplicate.

### 2.4. Antioxidant Activity

The antioxidant properties of mango waste extracts were evaluated using two methods: the in vitro free radical scavenging activity using 1,1-diphenyl-2-picrylhydrazyl (DPPH•), and the antioxidant effect against the lipid oxidation in food products using a vegetable edible oil (VEO) and cooked beef homogenate (CBH).

#### 2.4.1. DPPH• Scavenging Activity

The DPPH• scavenging activity was determined by following the procedure previously described by Castro-Vargas et al. [26]. As such, 50 μL of each extract solution (20 mg/mL) was added to 1.0 mL of 0.1 M DPPH• solution in ethanol (with initial absorbance measured at 517 nm “Ai”). The mixture was stirred and after 30 min at room temperature the final absorbance at 517 nm “Af” was measured. The radical scavenging percentage was determined as follows:

$$\%DPPH\;radical\;scavenging\;activity=\;\left(\frac{Ai-Af}{Ai}\right)\times 100$$

The radical scavenging properties of extracts were compared with the natural phenolic antioxidant gallic acid. All measurements were performed by triplicate and the results are presented as µmol Trolox equivalent per 100 g of raw material (µmol Trolox/100 g).

#### 2.4.2. Antioxidant Activity in Food Products

The antioxidant effect of extracts obtained from mango waste against lipid oxidation in food products was evaluated using VEO and CBH. Different batches of VEO and CBH were used in tests in order to obtain representative results regarding the extracts’ antioxidant properties. In addition, the AA of the extracts was compared to reference antioxidant compounds such as the commercial synthetic antioxidant tert-butylhydroquinone (TBHQ), and the natural phenolic antioxidant gallic acid. The lipid oxidation in the VEO and CBH samples was followed by measuring hydroperoxides and thiobarbituric acid reactive substances (TBARS).

The VEO sample (without antioxidants) was a mixture of edible oils (palm, soybean, and sunflower) for which the general composition was 70% unsaturated fatty acid triglycerides (40% oleic, 55% linoleic, and 5% of others), and 30% stearic acid triglycerides. The AA experiments were performed following the procedure previously described by Castro-Vargas et al. [27], consisting of two steps. The first was the accelerated oxidation of VEO samples with ferrous ion added as a pro-oxidant (3.5 mg/kg of oil), and each dry extract or the reference compounds (TBHQ or gallic acid) as antioxidants (300 mg/kg of oil). One VEO sample was prepared by applying the same procedure but without the addition of antioxidants, and was used as control sample. Then, all VEO samples were kept in oxidation for 15 days at 60 °C and every 12 hours these were saturated with oxygen gas. The second step was following the hydroperoxide and TBARS formation in all VEO samples at 0, 3, 6, 9, 12, and 15 days of oxidation. The hydroperoxides were measured by the conjugated dienes method, while the TBARS were analyzed using their absorbance at 532 nm; these were determined as described by Castro-Vargas et al. [27]. The concentration of hydroperoxides and TBARS in VEO samples was expressed as mmol of linoleic acid hydroperoxides equivalents per kg of oil, and as mg of malondialdehyde (MDA) per kg of oil, respectively. All experiments were performed by six replicates.

The AA experiments using CBH were performed following the procedure described by Castro-Vargas et al. [28]. The beef that was used was chuck cut, acquired from local markets in Bogotá (Colombia); 30 kg of the meat sample was randomly sampled. The sample was washed with water and trimmed of connective tissue and external fat; then, it was divided by successive quartering obtaining 48 fractions of 500 g approximately, and each fraction was cut into cubes and randomly mixed. The average fat content of the meat sample was 30 ± 3% (fresh weight). The beef samples were ground using a blender to obtain the meat homogenate (MH), and then individual portions of MH (approximately 100 g) were placed into plastic bottles and added with the dry extracts or reference antioxidants at 200 mg per kg of homogenate. The mixtures MH-extract or MH-antioxidant were cooked into a water bath at 75 °C until the internal temperature reached 65 °C. After cooking, the CBH were removed from the plastic bottles, the meat juice was drained, and the CBH were individually packaged into oxygen permeable bags. Finally, the CBH samples were stored for 9 days at 4 °C. A control sample was prepared following the same procedure; however, the extracts or the reference antioxidants were not added. The lipid oxidation of the CBH was evaluated at 0, 3, 6, and 9 days of storage by measuring hydroperoxides and TBARS by using the methods cited above and the procedure described by Castro-Vargas et al. [27]. The hydroperoxide concentrations are presented as mmol of linoleic acid hydroperoxides equivalents per kg of meat homogenate, and the TBARS concentration are expressed as mg of MDA per kg of meat homogenate. All experiments were performed by six replicates.

### 2.5. Antiproliferative Activity

The antiproliferative effect of mango waste extracts against some cell lines of human cancer were explored. The cell culture and antiproliferative activity measuring was developed according to the methodology described by Escobar et al. [29,30]. 

#### 2.5.1. Cell Lines, Culture, and Extract Preparation

Four cell lines were used: A-549 (human lung adenocarcinoma cells), HT-29 (human colorectal adenocarcinoma cells), MDA-MB-231 (human breast adenocarcinoma cells), and PC-3 (human prostate cancer cells). The cells were maintained with RPMI-1640 medium (Sigma-Aldrich) supplemented with 10% fetal bovine serum (Gibco), and antibiotics (100 U/mL penicillin and 100 μg/mL streptomycin, Gibco). The temperature was 37 °C in humified atmosphere with 5% carbon dioxide.

The extracts were dissolved in dimethyl sulfoxide (DMSO) at an initial concentration of 125 mg/mL; then, three serial dilutions were prepared from this stock solution: 125, 12.5, and 1.25 μg/mL. On the other hand, Taxol^®^ (paclitaxel, White Sulphur Springs, WV, USA) was used as a positive control at a concentration range between 0.5 and 50,000 nM. Additionally, the innocuous effect of DMSO on cells was evaluated using the same dilution range cited for the extracts. All treatments (extracts, Taxol^®^, and DMSO) were prepared in a culture medium, and the final concentration of DMSO for each treatment did not exceed 0.1% *v*/*v*.

#### 2.5.2. Resazurin Reduction Assay

Resazurin reduction assay was performed to assess the antiproliferative effect of the extracts. Briefly, the cells in the exponential growth phase were trypsinized (0.025% trypsin and 0.03% EDTA for 5 min at 37 °C), counted in a Neubauer chamber using the Trypan blue exclusion method, and seeded in 96-well flat-bottom microtiter plates at a density of 3500 cells per well. The plates were incubated for 24 h at 37 °C to allow adhesion to the support. After this period, the cell monolayer was exposed independently for 72 h at each treatment (extracts, Taxol^®^, and DMSO, at different concentration levels). After the incubation, the treatments were replaced by 100 µL of 44 µM resazurin prepared in culture media. The plates were incubated for 4 h at 37 °C; then, the fluorescence emitted by the viable or metabolically active cells was measured using an excitation wavelength of 535 nm and emission wavelength of 595 nm (TECAN GENios, Salzburg, Austria). For each cell line, a group of control cells was incubated using the same procedure but without addition of extracts, Taxol^®^ or DMSO. The fluorescence emitted by the treated cells was compared with the fluorescence emitted by the control cells, then, these were transformed to survival percentages. The experiments were performed during three different weeks, each week nine replicates of each treatment were made. The results of the antiproliferative activity are presented as cell growth percentage relative to control cells, in addition, the cellular response was observed as a function of the concentration of extracts, Taxol^®^, and DMSO.

### 2.6. Analysis of Phenolic Compounds

One extract with the best qualities (antioxidant and antiproliferative activities) was selected and analyzed by HPLC–ESI-MS, in order to identify some phenolic compounds. The analyses were performed using an Agilent Technologies 1260 chromatograph (Palo Alto, CA, USA) equipped with a binary pump (G1312B), a degasser, an autosampler, and a thermostatted column compartment. The extract was dissolved in methanol at 20 mg/mL and the injected sample was of 1 μL. Chromatographic separation was performed on a Luna Phenyl-hexyl column (Phenomenex 150 mm × 4.6 mm × 5 μm) kept at 30 °C. The mobile phase was composed of 0.1% formic acid (A) and acetonitrile (B) at a flow rate of 0.5 mL/min. The gradient program was as follows: 0% B (0 min), 15% B (20 min), 50% B (28 min), 70% B (30 min), 90% B (50 min), and 0% B (55 min); finally, the initial conditions were maintained for 10 min. The chromatographic system was coupled to a quadrupole time-of-flight mass spectrometer (MS) detector (Agilent Technologies 6520 Q-TOF, Palo Alto, CA, USA), equipped with an electrospray ionization (ESI) source operating in the negative ionization mode. The MS analysis settings were as follows: ESI capillary voltage, 4.0 kV; nitrogen as a drying and nebulizing gas at flow rate 10 L/min, temperature 350 °C, and pressure 40 psi; collimator voltage 175 V; and octopole voltage 750 V. The spectra were acquired over a mass to charge ratio (m/z) ranging from 50 to 1100. All results were analyzed using the MassHunter Workstation Agilent Technologies software. The phenolic compounds were identified using their mass spectra information, the molecular formula, and the bibliographic data.

### 2.7. Statistical Analysis

Statistical analyses were performed using the R (version 3.3.3, R Foundation for Statistical Computing, Vienna, Austria) and Statgraphics Centurion XVII software with a confidence level of 95%. The results are presented as mean and standard deviation. The statistical differences between mean values were stablished by one-way analysis of variance (ANOVA) and the Tukey’s test.

## 3. Results and Discussion

The waste (peels and seeds) generated from the agroindustrial processing of two Colombian mango cultivars (‘sugar mango’ and ‘Tommy Atkins’) were explored as source of bioactive phenolic compounds with antioxidant and antiproliferative properties. Phenolic extracts from mango waste were obtained, and their free radical scavenging activity and protective effect against lipid oxidation in food products were evaluated. In addition, their antiproliferative effects against four cell lines of human cancer were accessed.

### 3.1. Total Phenolic and Total Flavonoid Contents

Phenolic compounds are a big group of phytochemicals widely distributed in plants; they are well known for their beneficial biological effects and promising uses in food, cosmetic, and pharmaceutical products. The total phenolic content (TPC) and total flavonoid content (TFC) values present in the extracts that were obtained from the mango agroindustrial waste were evaluated and correlated with their antioxidant and antiproliferative properties. Table 1 shows the TPC values expressed as mg of gallic acid equivalents per 100 g of raw material (mg GAE/100 g). The highest TPC was observed in the ‘Tommy Atkins’ mango peel (PT), followed by the ‘sugar mango’ peel (PS), whereas the lower TPC values were observed in the ‘sugar mango’ seed kernel (SKS) and the ‘Tommy Atkins’ seed kernel (SKT). Both ‘sugar mango’ and ‘Tommy Atkins’ presented higher TPC in their peels and lower TPC in their kernels. These results are in line with previous reports. Ruales et al. [1] observed that the peel of Ecuadorian ‘Tommy Atkins’ mango contains 10 times more TPC compared with its seed kernel. However, studies with other varieties of mango showed a higher TPC in the kernels compared with the peels. Ribeiro et al. [5] reported from Brazilian mango cultivar ‘Ubá’ 8.25% and 5.72% TPC in the kernel and peel, respectively, while Prakash et al. [17] reported 5.96% and 3.59% TPC, respectively, from Indian mango varieties. These results suggest that the distribution of phenolic compounds in the mango agroindustrial waste (peels or seeds) can be affected by the mango cultivar. On the other hand, Table 1 presents the TFC values expressed as mg of quercetin per 100 g of raw material (mg quer/100 g). It is observed that the SKS and SKT presented higher TFC, followed by the PT and PS, whereas mango seed coats (SCS and SCT) showed lower TFC values. Considering all waste obtained from each mango cultivar (peel + coat + kernel), it is possible to observe that ‘Tommy Atkins’ has a greater TPC (total TPC = 4414.9 mg GAE/100 g), while ‘sugar mango’ has more TFC (total TFC = 83.4 mg quer/100 g).

Mango is one of the most widely distributed fruits in the world; many cultivars have been reported in tropical and subtropical countries of Africa, Asia, and Central and South America. In Colombia, ‘sugar mango’ and ‘Tommy Atkins’ are the most important varieties for the agroindustry [3]. The TPC values in kernels of different mango varieties from Asia and South America have been reported at between 1571 and 29,200 mg GAE/100 g [5,17,18]; the highest TPC was observed in the ‘Chok-Anan’ cultivar from Thailand (29,200 mg GAE/100 g) [18] and ‘Ubá’ from Brazil (8254 mg GAE/100 g) [5]. Studies on the phenolic composition of mango peels have reported TPC values between 28.5 and 5724 mg GAE/100 g [5,14,15,16,17]; the highest value was observed in the ’Small Tainong’ cultivar from China [31]. In ‘Tommy Atkins’ mango, Sogi et al. [6] reported TPC values in peel of between 2032 and 3185 mg GAE/100 g, and in kernel between 11,228 and 20,034 mg GAE/100 g. It is worth mentioning that previous reports on TPC in ‘sugar mango’ agroindustrial waste were not found. The TPC values observed in the present work for the mango peels are in the range that was previously cited, additionally, the TPC obtained for PT was higher than that reported by Sogi et al. [6] for Indian ‘Tommy Atkins’ cultivars. On the other hand, the content of phenolics observed for SKS and SKT were lower compared with the TPC above cited. The TPC values observed in the present study and reported in literature show significant variability, a difference that can be caused by the variations in the concentration of phenolic compounds in the mango fruits. Several reports indicate that the concentration and distribution of secondary metabolites in fruits (e.g., polyphenols) can be affected by parameters that are associated with their crop, post-harvest handling, and industrial processing [32,33].

### 3.2. Antioxidant Activity

Phenolic compounds are well known for their antioxidant activity (AA) related to degenerative and chronic disease prevention and the preservation of food products [34]. In this work, the antioxidant properties of phenolic extracts recovered from mango waste were evaluated by using the DPPH^•^ scavenging method. In addition, the efficiency of extracts to inhibit the lipid oxidation on food products was evaluated using vegetable edible oil (VEO) and cooked beef homogenate (CBH).

#### 3.2.1. DPPH• Scavenging Activity

Table 1 shows the results of the DPPH• scavenging expressed as µmol Trolox/100 g raw material. All extracts showed a lower radical scavenging activity compared to gallic acid, a natural phenolic antioxidant. For the extracts, the higher DPPH• scavenging activity was exhibited by the PT extract, followed by the PS extract; on the other hand, the lower DPPH• scavenging was observed by the kernel extracts. Of note, the TPC and DPPH• scavenging results show a correlation between the phenolic content in the extracts and their DPPH• scavenging properties, which is confirmed by a Pearson correlation analysis that indicates a high positive correlation (R^2^ = 0.951). A strong correlation between phenolic contents and radical scavenging properties has been observed and reported by extracts obtained from several vegetable products [35], including mango. Khammuang and Sarnthima [18] reported a high positive correlation (R^2^ = 0.953–0.962) between TPC and DPPH• scavenging properties of extracts obtained from mango seeds coat and kernel; similar results were observed by Huang et al. [14] for extracts recovered from various Taiwanese mango peels (R^2^ = 0.829–0.989). On the other hand, the Pearson correlation analysis shows a weak positive correlation (R^2^ = 0.678) between TFC values and DPPH• scavenging results. This weak correlation could partially be explained by the fact that not only the flavonoids are associated with the AA of the mango waste extracts; other phenolic compounds that are present could influence the results.

The AA by DPPH• scavenging method of the extracts from agroindustrial mango waste of ‘Tommy Atkins’ has been previously reported. Sogi et al. [6] observed values from 1310.70 to 1799.54 µmol Trolox/100 g for kernels, and values between 176 to 219 µmol Trolox/100 g for peels of Indian Tommy mango cultivars, while Ruales et al. [1] reported 44.34 µmol Trolox/100 g for kernels, and 11.06 µmol Trolox/100 g for peel of Ecuadorian ‘Tommy Atkins’ mango. The DPPH• scavenging activity observed in the present work for PT was higher compared to that reported by Sogi et al. and Ruales et al.; additionally, the AA observed for SKT was higher compared with the study by Ruales et al. Previous reports on radical scavenging activity of ‘sugar mango’ agroindustrial waste were not found; however, Corrales-Bernal et al. [4] explored the DPPH• scavenging capacity of ‘sugar mango’ flesh, and reported values from 5523 to 5907 µmol Trolox/100 g of flesh. The results obtained in the present study show that the agroindustrial waste from Colombian Tommy and ‘sugar mango’ cultivars are source of phenolic compounds with good free radical scavenging properties. This is noteworthy since this work could be considered the first report on the TPC, TFC, and DPPH• scavenging activity of ‘sugar mango’ agroindustrial waste.

#### 3.2.2. Antioxidant Activity in Food Products

Mango waste extracts were explored as natural antioxidants against lipid oxidation on VEO and CBH, and their effectiveness was compared with the TBHQ (as commercial synthetic antioxidant), and the gallic acid (as natural phenolic antioxidant). The antioxidant effect of mango waste extracts and reference antioxidants was evaluated by measuring the hydroperoxides and TBARS during different oxidation periods. Table 2 and Table 3 show the AA results in VEO and CBH, respectively. Hydroperoxides are expressed as mmol of linoleic acid hydroperoxides equivalents/kg, and TBARS are presented as mg of malondialdehyde/kg. All extracts (except SKT and PT extracts) and reference antioxidants delayed the formation of hydroperoxides and TBARS in VEO and CBH samples during the oxidation periods that were explored. The concentrations of oxidation products in the food samples added with the extracts and antioxidants were lower compared to the control sample. The extracts obtained from SKS and PS showed the highest antioxidant effect in VEO (compared with other extracts); moreover, their AA was similar compared with the reference antioxidants (see Table 2). Other extracts with AA in VEO were obtained from PT and SCT, specifically exhibiting a good antioxidant effect on the formation of TBARS. The formation of hydroperoxides in VEO samples was notably inhibited by both SKS and PS extracts. This behavior was observed during the 15 days of accelerated oxidation. A similar result was observed on the TBARS formation, in which case the antioxidant effectiveness of PS extract was higher. Compared to the control sample, SKS and PS extracts reduced the hydroperoxides and TBARS formation by more than 57% and 61%, respectively. These results were particularly evident between oxidation days 9 and 15.

Table 3 shows the AA results in CBH samples during their storage at 4 °C; PS, SKS, and SCT provided extracts with a high antioxidant effect on the hydroperoxides and TBARS formation. Of note, these extracts presented similar antioxidant effectiveness. This behavior was more notorious after 3 days of storage. Compared with the reference antioxidants, the PS, SKS, and SCT extracts showed a better AA than that of the gallic acid; besides, their antioxidant effects were close to those that observed for TBHQ. After 9 days of storage, the PS, SKS, and SCT reduced the hydroperoxides and TBARS formation in the CBH samples by more than 72% and 67%, respectively, considering that the higher concentrations of these substances were observed on the ninth day of storage.

The AA results that were observed may be related with the content of phenolic compounds in the extracts. Table 1 shows that some extracts with high AA in food products such as PS and SCT presented a good TPC. These results are in line with the ones reported previously by Torres-León et al. [20] and Siriamornpun et al. [21]. They observed that the lipid oxidation in food products was efficiently inhibited by the extracts obtained from mango waste (seed kernels and peels) with high content of phenolic compounds. Another extract with high AA in VEO and CBH was SKS; however, its antioxidant properties cannot be correlated with their TPC. For this extract, the AA is probably related with the content of flavonoids; the TFC value of SKS was the highest observed in the present study. Flavonoids recovered from several natural sources have been associated with the inhibition of lipid oxidation in food products such as salmon fillets [36], pork slices [37], chicken pâtés [38], and cottonseed oil [39]. 

Lipid oxidation in foods is a complex process, which can be developed in several ways and by means of different reaction mechanisms. The oxidation of polyunsaturated fatty acids can generate a wide range of products: hydroperoxides, aldehydes, ketones, esters, ethers, short-chain acids, alcohols, lactones, furans, and aliphatic and aromatic hydrocarbons [40]. The hydroperoxides are considered as initial lipid oxidation products, their degradation, and additional secondary reactions, generate the intermediate and final products [40]. Some intermediate and final products, such as aldehydes, ketones, and organic acids, are known as thiobarbituric acid reactive substances (TBARS) [41]. Lipid oxidation products are related with food spoilage, since these substances change their nutritional value and organoleptic properties, moreover, they reduce quality and shelf life. A suitable antioxidant for food products should reduce/inhibit the formation of lipid oxidation products in its initial, intermediate, and final stages, in order to avoid/minimize food spoilage and its hazardous effects on consumers. The results observed in this study show that the phenolic extracts obtained from mango waste, particularly SKS and PS extracts, inhibit the lipid oxidation in VEO and CBH during its different stages; these extracts showed a good efficacy to reduce the formation of hydroperoxides (initial oxidation products) and the TBARS (secondary/final products). Hence, PS and SKS could be considered as promising natural sources of food preservatives, furthermore, an alternative to replace the synthetic antioxidants used for some food industries (e.g., TBHQ). Previous reports show that mango seed kernels are source of phenolics with protective effects against lipid oxidation on buffalo butter fat [42] and oil/water emulsion [17], while phenolics from mango peels have antioxidant effects on rice flour oxidation [21].

### 3.3. Antiproliferative Activity

**Figure 2:**
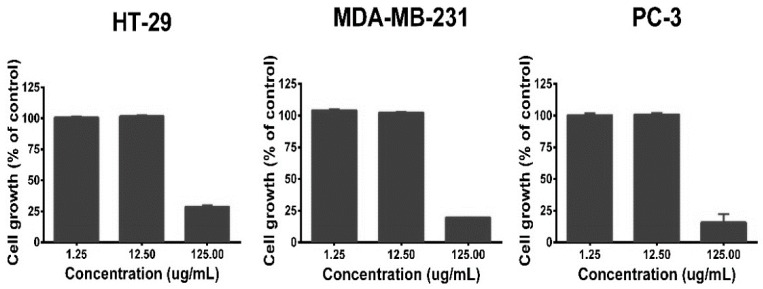
Antiproliferative activity of the extract obtained from the ‘sugar mango’ seed coat. Cell lines: HT-29 (colorectal adenocarcinoma), MDA-MB-231 (breast adenocarcinoma), and PC-3 (prostate cancer).

**Figure 3:**
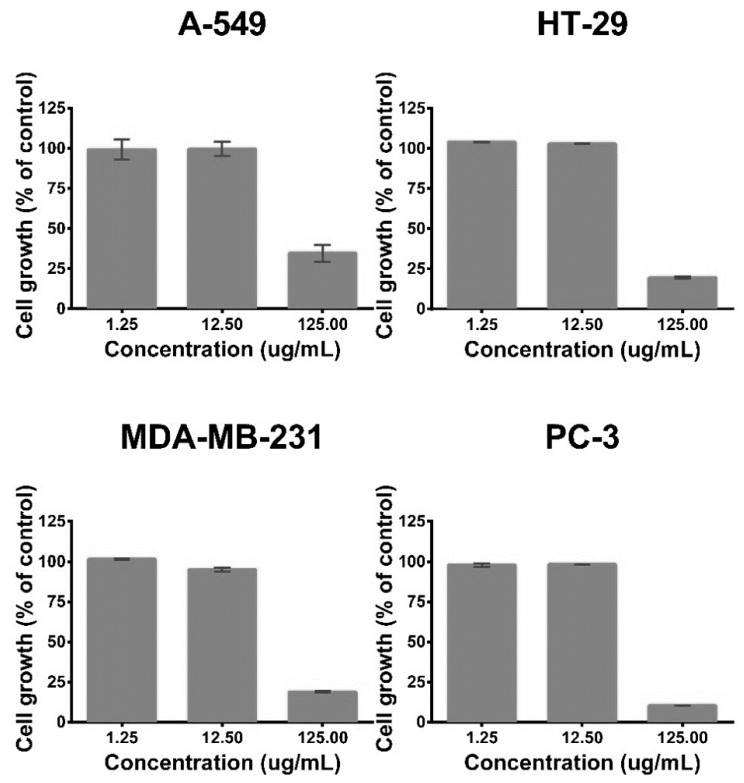
Antiproliferative activity of the extract obtained from the ‘sugar mango’ seed kernel. Cell lines: A-549 (lung adenocarcinoma), HT-29 (colorectal adenocarcinoma), MDA-MB-231 (breast adenocarcinoma), and PC-3 (prostate cancer).

The results presented in the previous section show that the agroindustrial mango waste is a source of phenolic compounds with antioxidant properties. These substances are also recognized by their beneficial biological effects, including the anticancer activity [34,43]. In this study, the antiproliferative effect of the extracts against some cell lines of human cancer were explored, four cell lines were used: A-549 (human lung adenocarcinoma line cell), HT-29 (human colorectal adenocarcinoma line cell), MDA-MB-231 (human breast adenocarcinoma line cell), and PC-3 (human prostate cancer line cell). Initially, the effect of the DMSO (used as a vehicle for extract preparation) on cell viability was evaluated; any interference in the antiproliferative results was observed considering the DMSO concentration that was used (0.1% *v*/*v*). Also, the cell sensitivity of the cell lines was evaluated using Taxol^®^ (positive control), this substance is an antineoplastic agent that is clinically used to treat patients with lung, ovarian, and breast cancer, and advanced forms of Kaposi’s sarcoma. The results of the cell line sensitivity against Taxol^®^ (at different concentrations) are shown in Figure S1 (see Supplementary data); all cell lines showed a concentration-dependent reduction of their viability, and HT-29 exhibited higher sensitivity, followed by MDA-MB-231 and PC-3. On the other hand, the antiproliferative activity experiments using the mango waste extracts showed that only the ones obtained from SKS and SCS have an effect on the cell viability. Figure 2 and Figure 3 show the results of the antiproliferative activity of SCS and SKS extracts, respectively. The SKS extract exhibited antiproliferative effects against all cell lines, while the SCS extract showed an effect against HT-29, MDA-MB-231, and PC-3. The activity observed can be considered as moderate, due to the extracts showed effects on cell lines only at their highest concentration (125 mg/mL). The PC-3 cell line presented the higher sensitivity to SKS extract; its cell growth was reduced more than 80%, whereas MDA-MB-231 and HT-29 showed a cell growth reduction lower than 75%. The SCS extract exhibited the same effect on the HT-29, MDA-MB-231 and PC-3 cell lines; their viability was decreased by 75%. The results obtained in the present work are in line with previous reports about the antiproliferative activity of extracts obtained from mango seed kernels; Abdullah et al. [22] observed that ethanolic extracts exhibit antiproliferative effects against breast cancer cell lines (MCF-7 and MDA-MB-231); in addition, they observed that the extracts do not have toxicity in normal breast cells (MCF-10A).

The mango has been identified as a source of extracts and compounds with antiproliferative activities. These properties have been related with the phenolic compounds present in mango fruit (peel, flesh, and seed) [43]; phenolic acids, flavonoids (e.g., mangiferin), and galatotanins are noteworthy due to their high bioactivity [23,44,45]. Previously, Noratto et al. [46] evaluated the antiproliferative potential on several cancer cell lines (breast cancer MDA-MB-231, leukemia Molt-4, lung cancer A-549, prostate cancer LnCap, and colon cancer SW-480) of the phenolic extracts obtained from the flesh of different mango cultivars. They observed that the phenolics in the extracts were associated with an increase in the mRNA expression of the pro-apoptotic and cell-cycle regulator biomarkers, as well as a decrease in the generation of reactive oxygen species (antioxidant activity). In the present work, the antiproliferative activity observed for SKS extract may be related with its high flavonoid content (see Table 1), in addition, Table 4 shows that mangiferin, a promising anticancer flavonoid [23,45], was identified in SKS extract. Other phenolics with antiproliferative activity are the gallic acid derivates (e.g., galloyl glucosides) [22]. These compounds were also observed in SKS extract (see Table 4) and their possible effects on cancer cell lines will be explained in the following section.

### 3.4. Analysis of Phenolic Compounds

The results described above indicate that the waste generated from the agroindustrial processing of Colombian mango cultivars, ‘sugar mango’ and ‘Tommy Atkins’, are sources of bioactive phenolic compounds with antioxidant and antiproliferative properties. ‘Sugar mango’ waste provided extracts with free radical scavenging activity, a preservative effect against lipid oxidation in food products, and antiproliferative properties on several cancer cell lines. SKS supplies a phenolic extract with the best antioxidant and antiproliferative activities. Considering the aforementioned, the SKS extract was selected and analyzed by HPLC-ESI-MS; some phenolic compounds were tentatively identified, and these were related with the antioxidant and antiproliferative results. Table 4 shows the chromatographic and mass spectra information of the phenolic compounds, tentatively identified in the SKS extract; furthermore, Figures S2 and S3 (see supplementary material) show the HPLC-ESI-MS profile of extract and mass spectra of detected compounds, respectively. Twenty-two signals were considered in the chromatographic profile of the SKS extract, and fourteen phenolic compounds were identified (see Figure S2 and Table 4): four phenolic acids, eight galloyl glucosides, one gallic acid ester, and one flavonoid. Shikimic acid has been previously reported in mango flesh of the Ubá cultivar from Brazil [5], compound that is a precursor of different phenolics, particularly flavonoids. Galloyl glucose, 5-O-galloylquinic acid, and methyl digallate ester were observed in flesh, peels, and kernels of ‘Keitt’, ‘Osteen’, and ‘Sensación’ mango cultivars from Spain, whereas galloyl diglucoside was observed only in peels and kernels of the same cultivars [47,48]. Mangiferin is the most predominant flavonoid in mango, and it has been detected in mango stem bark, peel, flesh, kernel, and leaves from several mango cultivars (e.g., ‘Tommy Atkins’, ‘Ubá’, ‘Keitt’, ‘Osteen’, ‘Sensación’, ‘Van Dyke’, and ‘Embrapa-141-Roxa’) [1,5,47,48,49]. The polygalloyl glucosides are a group of phenolics derived from the gallic acid that is widely distributed in mango, previous reports cite from trygalloyl glucosides until nonagalloyl glucosides. The tetragalloyl glucose has been reported in peels and kernels [49], while the pentagalloyl glucose was observed in flesh, peels, and kernels [47,48]. The concentration of mangiferin in kernels from various cultivars has been reported between 0.05 and 6.4 mg/g [1,5,49], the higher content was observed in the Brazilian cultivar ‘Van Dyke’. Additionally, in this last cultivar the tetragalloyl glucose content was observed at 0.99 mg/kg. It is remarkable that in Section 3.1 we discussed that the concentration of secondary metabolites in fruits can be affected by several parameters; Vithana et al. [50,51,52] show that the harvest maturity stage, the pre-harvest processing, and post-harvest storage affect the concentration of mangiferin and phenolic acids in all mango fruit parts.

The phenolic compounds observed in SKS extracts can be related with their antioxidant and antiproliferative properties. In general, phenolics are recognized by their high potential to transfer electrons and hydrogen atoms to free radical and reactive oxygen species; moreover, their final products after the reduction–oxidation reaction are frequently chemically stable. Mangiferin is the most recognized bioactive phenolic compound in mango; its antioxidant activity in biological systems and food products has been previously explored. Several studies suggest that mangiferin helps to delay the lipid oxidation induced by pro-oxidant ions such as iron (Fe^2+^/Fe^3+^) in biological systems and food products (e.g., meat products) [25]. This effect is due to the fact that the mangiferin catechol moiety forms a strong complex with iron ions, thereby; it prevents the generation of hydroxyl radical in fenton-type reactions. Additionally, in the mangiferin–iron complex, the iron improves its superoxide radical scavenging action. Recently, Silva da Veiga et al. [53] described the structure–antioxidant relationship of mangiferin using quantum chemistry calculations; they conclude that mangiferin has greater antioxidant capacity by hydrogen transfer than by electron transfer. Furthermore, a synergistic effect can be observed between xanthone and the sugar rings. Mangiferin and its extracts have been explored as antioxidant and functional additives for food industries. Imran et al. [54] used the agroindustrial mango waste as source of mangiferin to prepare functional drinks, while Boonnattakorn et al. [55] added mangiferin to ethylene vinyl acetate matrix in order to obtain an antioxidant packaging for food products. On the other hand, mangiferin is a recognized anticancer compound from mango; its properties have been reported and discussed by several studies. Gold-Smith et al. [23] presented a complete review on the potential role and action mechanisms of mangiferin against cancer. They described that mangiferin has potent antioxidant and anti-inflammatory effects, causing cell cycle arrest, reducing proliferation and metastasis, promoting apoptosis in malignant cells, and protecting the cells and biomolecules (e.g., DNA) against oxidative stress and damage. The antiproliferative effect of mangiferin is considered promising to reduce the malignant tumor volume, this compound has shown a similar efficiency compared to cisplatin. In addition, mangiferin exhibits low toxicity (it has a broad intake safety margin), and has not shown cytotoxic effect on normal cells; thus, mangiferin will be a candidate for cancer therapies.

Gallic acid derivates such as galloyl glucosides have been associated with the antioxidant and antiproliferative activities of mango peels and kernel extracts; Torres-León et al. [20] reported pentagalloyl glucoside as the major phenolic compound identified in mango seed kernel (‘Ataulfo’ cultivar from Mexico), and suggests that it is related with the DPPH• scavenging activity of kernel extracts. Additionally, Jiang at al. [56] reported that the pentagalloyl glucoside from mango peels possessed potent scavenging effects on hydroxyl radicals, superoxide anions, and singlet oxygen. Namngam et al. [57] isolated different phenolic fractions from kernels of ‘Kaew’ and ‘Choke-Anan’ cultivars from Thailand. They observed that the rich fraction of galloyl glucosides (particularly tetragalloyl glucoside and hexagalloyl glucoside) exhibited the highest free radical scavenging activities as well as inhibitory effects against oxidation process mediated by enzymes. The galloyl glucosides also exhibited antiproliferative effects against breast cancer (MDA-MB-231), liver cancer (HepG2), and leukemia (HL-60). These properties have been associated with their antioxidant activity, which acts on the inflammatory mechanisms that are associated with cancer development [20,22]. Finally, the interaction effects (additivity, synergism, and antagonism) between phenolic compounds have been studied. Rocha et al. [44] reported that the mixtures of hydroxycinnamic acids, hydroxybenzoic acids, and their derivatives, exhibit important effects on the induction of differentiation and cellular apoptosis in colon, liver, prostate, breast, and lung tumor cell lines. Based in previous reports, it is possible to suggest that the phenolics that are observed in SKS extract contributed significantly to its antioxidant and antiproliferative properties. Mangiferin and galloyl glucosides are valuable phenolics that are present in SKS with functional properties and promising uses in food and pharmaceutical products.

## 4. Conclusions

The agroindustrial waste from the Colombian mango cultivars ‘sugar mango’ and ‘Tommy Atkins’ was explored for sources of bioactive phenolic compounds. The results obtained showed that phenolic compounds from mango waste are related with the antioxidant and antiproliferative activities observed. Peels provide extracts with high total content of phenolics, while kernels are sources of flavonoids. All mango waste studied contained phenolics with free radical scavenging activity. ‘Sugar mango’ waste supplied extracts with antioxidant effect in food products and antiproliferative properties; the best qualities were exhibited by the extract from the ‘sugar mango’ kernel. Fourteen phenolic compounds were identified in the extract from the ‘sugar mango’ kernel; mangiferin, and galloyl glucosides are recognized as bioactive substances from mango kernels. The agroindustrial waste from sugar mango, especially its kernel, may be considered a good source of bioactive phenolic compounds, with promising uses in food and pharmaceutical products. This thereby represents an alternative use for mango waste, giving it added value (valorization), and helping to reduce its environmental effects. However, additional studies are necessary in order to establish the most suitable recovery method as well as the bioavailability and safety of bioactive compounds from mango waste. Finally, the present work could be considered as the first report on bioactive phenolics from the Colombian ‘sugar mango’ cultivar.

## Figures and Tables

**Table 1:** Total phenolic content, total flavonoid content, and DPPH radical scavenging of the extracts obtained from mango agroindustrial waste.

Mango Waste	TPC (mg GAE/100 g Raw Material)	TFC (mg quer/100 g Raw Material)	DPPH Radical Scavenging Activity (µmol Trolox/100 g Raw Material)
PS	1838.3 ± 0.01 ^b^	19.8 ± 0.01 ^d^	4480 ± 3 ^c^
SCS	723.0 ± 0.14 ^c^	7.5 ± 0.03 ^e^	1970 ± 4 ^d^
SKS	48.6 ± 0.09 ^f^	56.1 ± 0.01 ^a^	40 ± 0.5 ^g^
PT	3553.2 ± 0.01^a^	26.0 ± 0.01 ^c^	6280 ± 10 ^b^
SCT	581.4 ± 0.01 ^d^	4.8 ± 0.01 ^f^	790 ± 6 ^e^
SKT	280.3 ± 0.01 ^e^	34.4 ± 0.01 ^b^	180 ± 2 ^f^
Gallic acid	-	-	27899 ± 65 ^a^

All columns show the means ± standard deviation (*n* = 3); According to the Tukey’s test, means in columns followed by the same letter (^a–h^) are not statistically different (*p* < 0.05). TPC: total phenolic content; TFC: total flavonoid content; PS: ‘sugar mango’ peel; SCS: ‘sugar mango’ seed coat; SKS: ‘sugar mango’ seed kernel; PT: ‘Tommy Atkins’ mango peel; SCT: ‘Tommy Atkins’ mango seed coat; SKT: ‘Tommy Atkins’ mango seed kernel; GAE: gallic acid equivalents.

**Table 2:** Antioxidant effect of the extracts obtained from agroindustrial mango waste, tert-butylhydroquinone (TBHQ), and gallic acid on the formation of hydroperoxides and thiobarbituric acid reactive substances in vegetable edible oil.

**Hydroperoxides ^1^**						
**Sample**	**Oxidation Time (days)**					
	**0**	**3**	**6**	**9**	**12**	**15**
Control ^2^	11.03 ^ax^	11.28 ^cx^	13.52 ^dx^	32.22 ^ay^	52.92 ^az^	54.69 ^az^
PS	5.18 ^cx^	11.00 ^cx^	12.44 ^ex^	15.02 ^ey^	16.22 ^fy^	23.35 ^ez^
SCS	5.55 ^cw^	7.40 ^dw^	15.55 ^dx^	19.70 ^cy^	22.90 ^ez^	26.15 ^dz^
SKS	5.06 ^cv^	8.80 ^dw^	12.23 ^ex^	13.86 ^fx^	17.43 ^fy^	23.18 ^ez^
PT	6.10 ^bv^	17.28 ^bw^	18.95 ^cw^	21.13 ^bx^	29.80 ^cy^	48.21 ^bz^
SCT	5.06 ^cw^	8.53 ^dx^	21.21 ^by^	23.47 ^by^	27.12 ^dz^	29.31 ^cz^
SKT	5.91 ^bv^	18.96 ^aw^	25.19 ^ax^	33.10 ^ay^	36.15 ^by^	46.53 ^bz^
TBHQ	5.55 ^cw^	10.23 ^cx^	19.86 ^cz^	17.89 ^dy^	18.00 ^fy^	18.18 ^gy^
Gallic acid	5.06 ^cw^	8.42 ^dx^	10.91 ^ex^	17.50 ^dy^	16.33 ^fy^	20.26 ^fz^
**Thiobarbituric Acid Reactive Substances ^3^**						
**Sample**	**Oxidation Time (days)**					
	**0**	**3**	**6**	**9**	**12**	**15**
Control ^2^	0.18 ^aw^	2.68 ^ax^	3.91 ^ay^	6.35 ^az^	6.05 ^az^	7.03 ^az^
PS	0.04 ^cv^	0.04 ^ev^	0.39 ^ew^	0.75 ^gx^	1.67 ^fy^	2.07 ^ez^
SCS	0.18 ^av^	1.82 ^bw^	2.46 ^bx^	4.26 ^by^	5.07 ^bz^	5.93 ^bz^
SKS	0.04 ^cw^	0.09 ^ex^	1.79 ^cy^	1.85 ^dy^	2.17 ^dz^	2.74 ^dz^
PT	0.04 ^cv^	0.83 ^dw^	1.26 ^cx^	1.89 ^dy^	2.39 ^dz^	2.05 ^ey^
SCT	0.04 ^cv^	0.16 ^ew^	1.32 ^cx^	1.52 ^ex^	1.94 ^ey^	2.83 ^dz^
SKT	0.04 ^cv^	0.85 ^cw^	1.37 ^cx^	1.73 ^dy^	2.20 ^dz^	2.07 ^ez^
TBHQ	0.18 ^aw^	0.40 ^dx^	3.06 ^ay^	2.94 ^cy^	3.28 ^cz^	3.67 ^cz^
Gallic acid	0.04 ^cw^	0.09 ^ex^	0.99 ^dy^	1.11 ^fy^	1.29 ^gy^	2.78 ^dz^

^1^ Expressed as mmol of linoleic acid hydroperoxides equivalents/kg of oil; ^2^ Control: sample without extracts or reference antioxidant compounds; ^3^ Expressed as mg malondialdehyde/kg of oil, all columns show the means (*n* = 6); ^a–g^ Means with different letters in a column are significantly different between extracts according to one-way ANOVA (*p* < 0.05); ^v–z^ Means with different letters in a row are significantly different between oxidation times according to one-way ANOVA (*p* < 0.05). PS: ‘sugar mango’ peel; SCS: ‘sugar mango’ seed coat; SKS: ‘sugar mango’ seed kernel; PT: ‘Tommy Atkins’ mango peel; SCT: ‘Tommy Atkins’ mango seed coat; SKT: ‘Tommy Atkins’ mango seed kernel.

**Table 3:** Antioxidant effect of the extracts obtained from agroindustrial mango waste, TBHQ, and gallic acid on the formation of hydroperoxides and thiobarbituric acid reactive substances in cooked beef homogenate stored at 4 °C.

**Hydroperoxides ^1^**				
**Sample**	**Oxidation Time (days)**			
	**0**	**3**	**6**	**9**
Control ^2^	5.49 ^aw^	11.04 ^ax^	23.54 ^ay^	32.07 ^az^
PS	5.38 ^ax^	6.91 ^dy^	6.55 ^ey^	8.12 ^cz^
SCS	5.15 ^aw^	10.73 ^az^	6.18 ^ex^	8.47 ^cy^
SKS	3.83 ^bx^	6.31 ^ey^	6.20 ^ey^	8.93 ^cz^
PT	4.24 ^bw^	7.55 ^dx^	9.70 ^cy^	10.85 ^bz^
SCT	3.83 ^bw^	6.95 ^dz^	6.42 ^ex^	7.30 ^dy^
SKT	4.24 ^bx^	7.49 ^dy^	10.11 ^bz^	10.66 ^bz^
TBHQ	5.15 ^ax^	4.76 ^fx^	5.51 ^ey^	6.93 ^dz^
Gallic acid	3.83 ^bx^	8.13 ^cy^	8.03 ^dy^	10.31 ^bz^
**Thiobarbituric Acid Reactive Substances ^3^**				
**Sample**	**Oxidation Time (days)**			
	**0**	**3**	**6**	**9**
Control ^2^	0.54 ^ax^	0.65 ^by^	0.71 ^by^	1.16 ^bz^
PS	0.17 ^dy^	0.14 ^dy^	0.31 ^dz^	0.28 ^gz^
SCS	0.33 ^bx^	0.35 ^cx^	0.44 ^cy^	0.61 ^dz^
SKS	0.22 ^cy^	0.36 ^cz^	0.32 ^dz^	0.38 ^fz^
PT	0.17 ^dx^	1.37 ^ay^	1.47 ^ay^	1.65 ^az^
SCT	0.22 ^cy^	0.30 ^cz^	0.19 ^ey^	0.25 ^gz^
SKT	0.17 ^dy^	0.67 ^bz^	0.67 ^bz^	0.70 ^cz^
TBHQ	0.13 ^ey^	0.12 ^dy^	0.37 ^cz^	0.37 ^fz^
Gallic acid	0.12 ^ex^	0.15 ^dx^	0.38 ^cy^	0.52 ^ez^

^1^ Expressed as mmol of linoleic acid hydroperoxides equivalents/kg of meat homogenate; ^2^ Control: sample without extracts or reference antioxidant compounds; ^3^ Expressed as mg malondialdehyde/kg of meat homogenate, all columns show the means (*n* = 6); ^a–g^ Means with different letters in a column are significantly different between extracts according to one-way ANOVA (*p* < 0.05); ^w–z^ Means with different letters in a row are significantly different between oxidation times according to one-way ANOVA (*p* < 0.05). PS: ‘sugar mango’ peel; SCS: ‘sugar mango’ seed coat; SKS: ‘sugar mango’ seed kernel; PT: ‘Tommy Atkins’ mango peel; SCT: ‘Tommy Atkins’ mango seed coat; SKT: ‘Tommy Atkins’ mango seed kernel.

**Table 4:** Phenolic compounds tentatively identified by HPLC–ESI-MS(Q-TOF) in the extract obtained from ‘sugar mango’ seed kernel.

Signal	Proposed Compound	Retention Time (Minutes)	[M−H]^−^m/z ^1^	Molecular Formula	Error (ppm)	Score (%)
1	3-β-Galactopyrasonyl glucose	3.998	341.1101	C_12_H_22_O_11_	−3.11	97.13
2	Shikimic acid	5.705	173.0451	C_7_H_10_O_5_	2.62	98.82
3	Quinic acid	6.732	191.0562	C_7_H_12_O_6_	0.42	99.47
4	Galloyl glucose	13.078	331.0676	C_13_H_16_O_10_	−0.89	99.36
5	Galloyl diglucoside	17.005	493.1203	C_19_H_26_O_15_	−0.42	99.11
6	5-O-galloylquinic acid	19.225	343.0679	C_14_H_16_O_10_	−1.93	97.18
7	NI	23.434	443.1933	-	−2.32	-
8	NI	26.002	183.0314	-	−1.29	97.21
9	Mangiferin	27.576	421.0784	C_19_H_18_O_11_	0.25	99.58
10	Trygalloyl glucose isomer I	28.421	635.0892	C_27_H_24_O_18_	0.25	99.58
11	Trygalloyl glucose isomer II	28.753	635.0895	C_27_H_24_O_18_	−1.92	96.54
12	Tetragalloyl glucose isomer I	29.068	787.1003	C_34_H_28_O_22_	0.13	99.50
13	Tetragalloyl glucose isomer II	29.383	787.0993	C_34_H_28_O_22_	−0.81	97.25
14	Tetragalloyl glucose isomer III	29.581	787.1003	C_34_H_28_O_22_	−0.45	99.36
15	Pentagalloyl glucose	29.929	939.1111	C_41_H_32_O_26_	0.91	93.54
16	Methyl digallate ester	30.675	335.0425	C_15_H_12_O_9_	0.51	99.06
17	NI	31.172	907.1197	C_41_H_32_O_24_	−1.34	97.26
18	NI	33.094	374.3275	-	−2.40	95.75
19	NI	33.889	416.3752	-	2.08	-
20	Anacardic acid	46.863	343.2284	C_22_H_32_O_3_	-	-
21	Ginkgoic acid	48.504	345.2447	C_22_H_34_O_3_	-	-
22	NI	49.001	371.2610	C_24_H_36_O_3_	−1.65	99.30

^1^ Experimental mass-charge relation of molecular ion [M−H]^−^; NI: not identified.

## Supplementary Materials

The following are available online at http://www.mdpi.com/2076-3921/8/2/41/s1. Figure S1. Cell line sensitivity against Taxol^®^. Figure S2. Chromatographic profile of SKS extract obtained by HPLC–ESI-MS(Q-TOF). Figure S3. Mass spectra of compounds detected in SKS extract by HPLC–ESI-MS(Q-TOF).
