# Supplementary material for: Bioactive Phenolic Compounds from the Agroindustrial Waste of Colombian Mango Cultivars ‘Sugar Mango’ and ‘Tommy Atkins’—An Alternative for Their Use and Valorization
Source: Antioxidants (Basel). 2019 Feb 15;8(2):41. doi: 10.3390/antiox8020041 (PMC6406469; doi:10.3390/antiox8020041)

**Figure S1.** Cell line sensitivity against Taxol^®^. A-549 (lung adenocarcinoma), HT-29 (colorectal adenocarcinoma), MDA-MB-231 (breast adenocarcinoma) and PC-3 (prostate cancer).


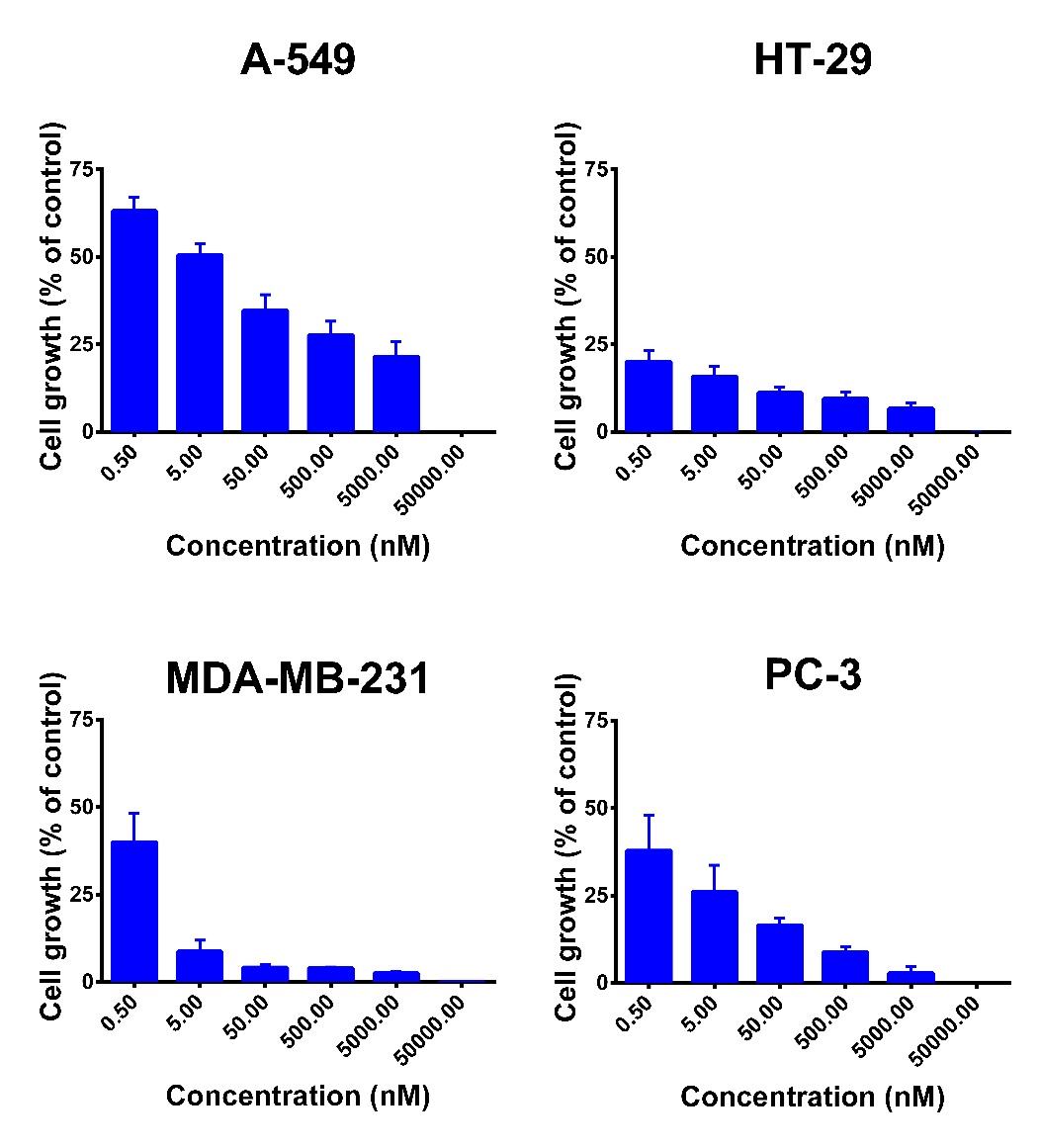

Supplement: Supplementary file 1 [file antioxidants-08-00041-s001.zip › Supplementary files/~WRL1625.tmp]
