# Supplementary material for: Bioactive Phenolic Compounds from the Agroindustrial Waste of Colombian Mango Cultivars ‘Sugar Mango’ and ‘Tommy Atkins’—An Alternative for Their Use and Valorization
Source: Antioxidants (Basel). 2019 Feb 15;8(2):41. doi: 10.3390/antiox8020041 (PMC6406469; doi:10.3390/antiox8020041)

Figure S3. Mass spectra of compounds detected in SKS extract by HPLC-ESI-MS(Q-TOF)

Signal 1: 3.998 min. 3-β-Galactopyrasonyl glucose ($C_{12}H_{22}O_{11}$)

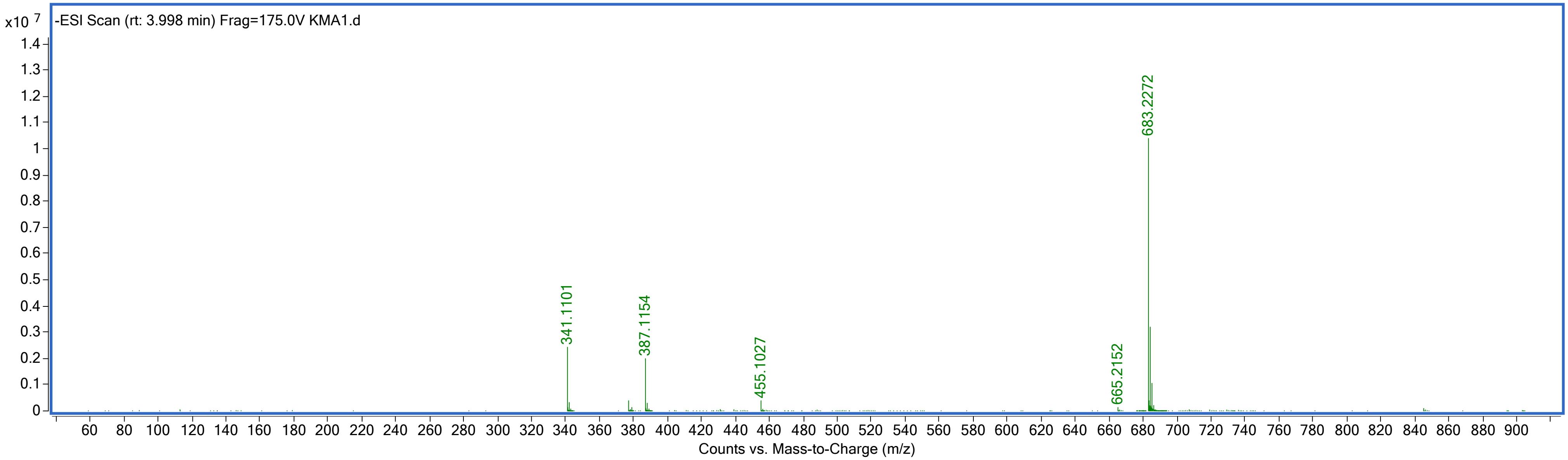

Signal 2: 5.705 min. Shikimic acid ($C_7H_{10}O_5$)

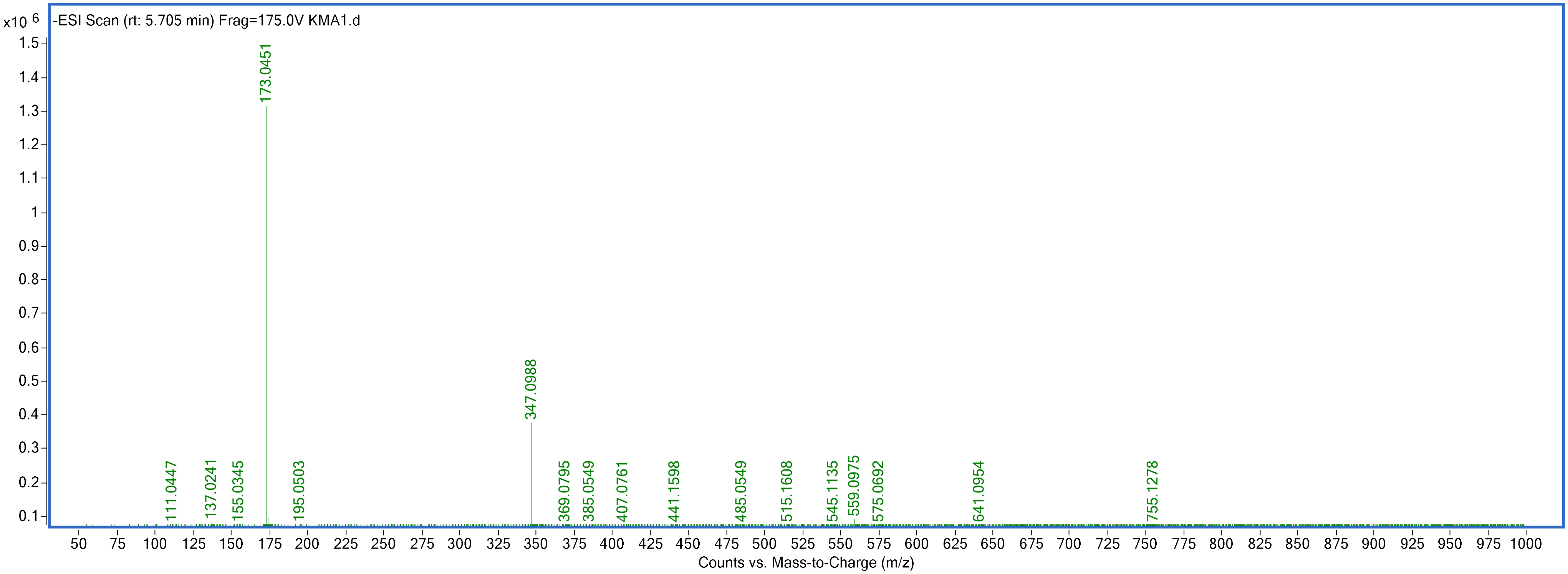

Signal 3: 6.732 min. Quinic acid ($C_7H_{12}O_6$)

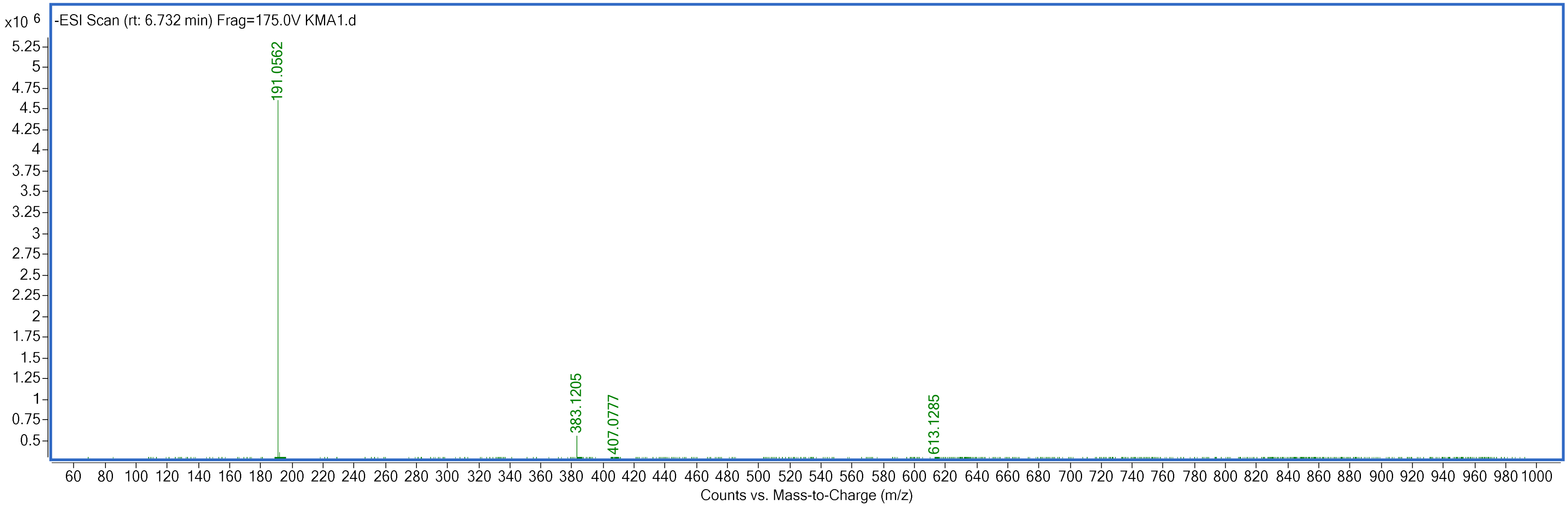

Signal 4: 13.078 min. Galloylglucose ($C_{13}H_{16}O_{10}$)

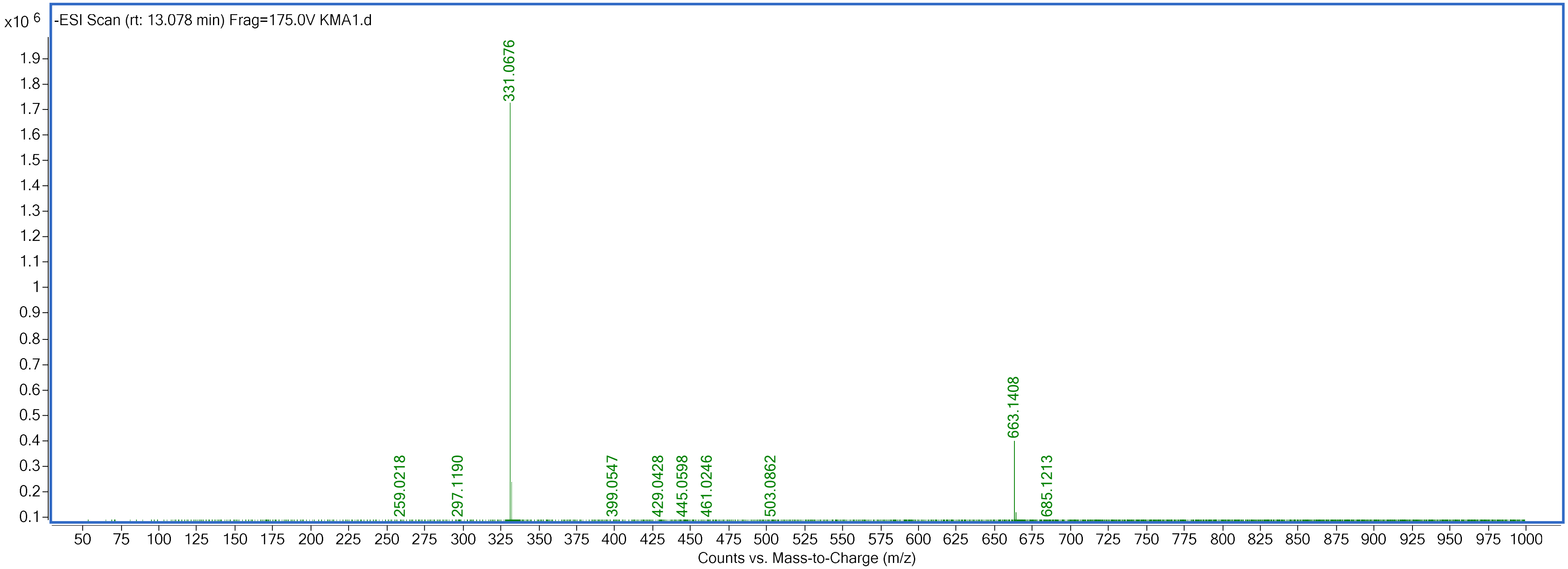

Signal 5: 17.005 min. Galloyl diglucoside ($C_{19}H_{26}O_{15}$)

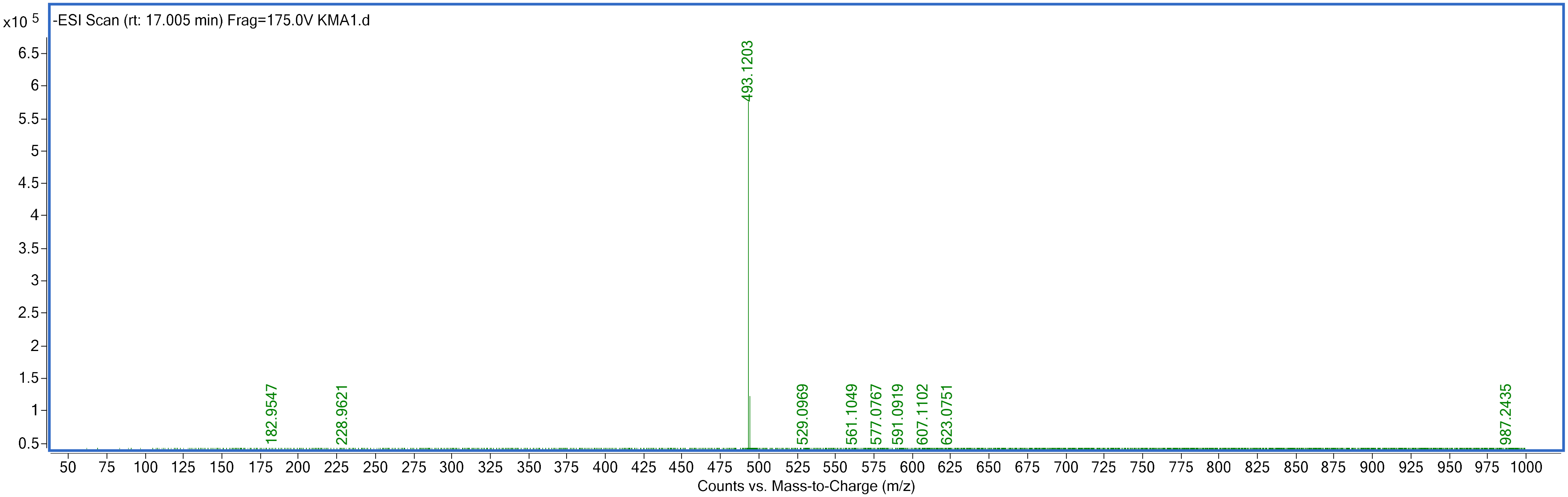

Signal 6: 19.225 min. 5-O-Galloylquinic acid ($C_{14}H_{16}O_{10}$)

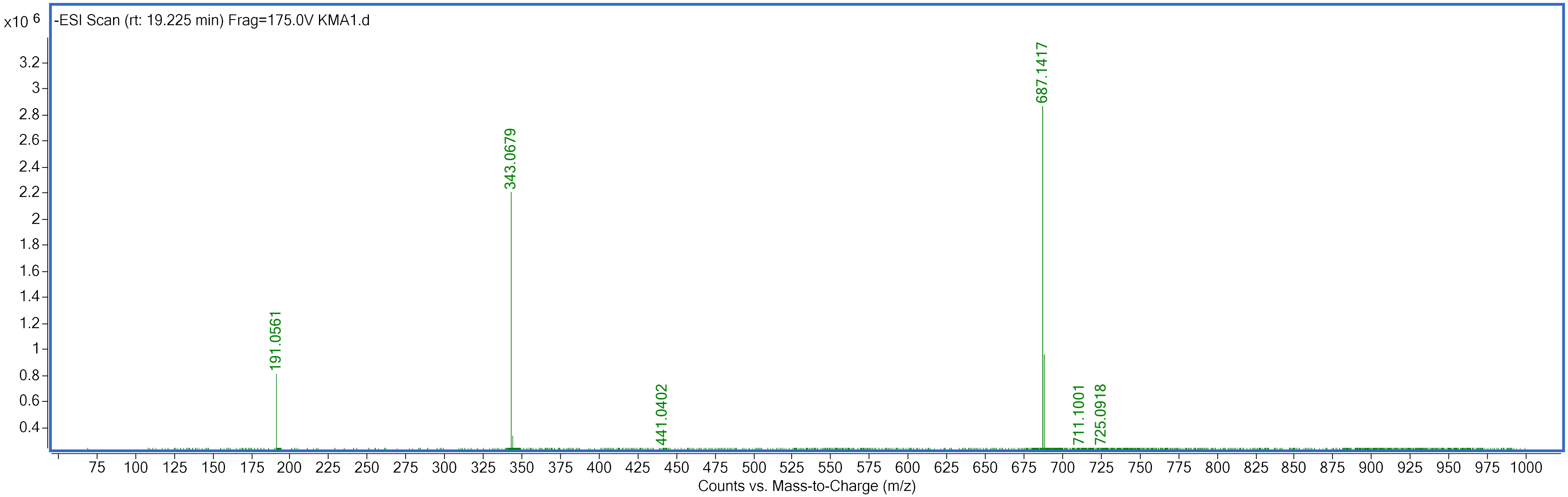

Signal 7: 23.434 min. NI

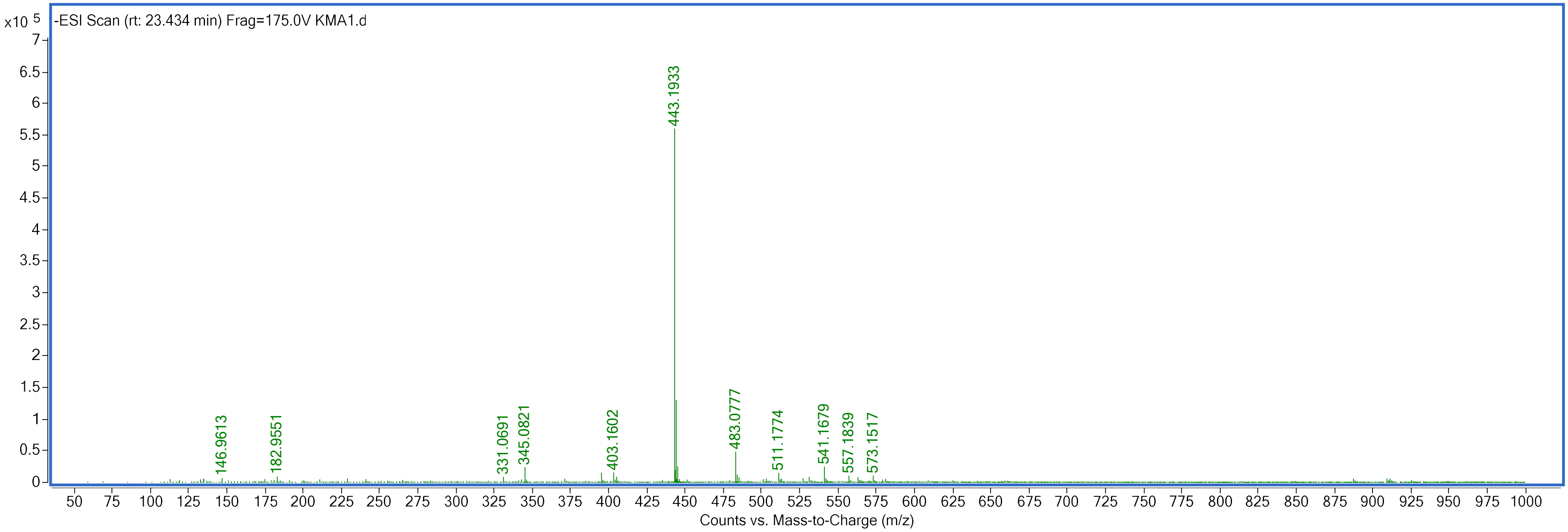

Signal 8: 26.002 min. NI

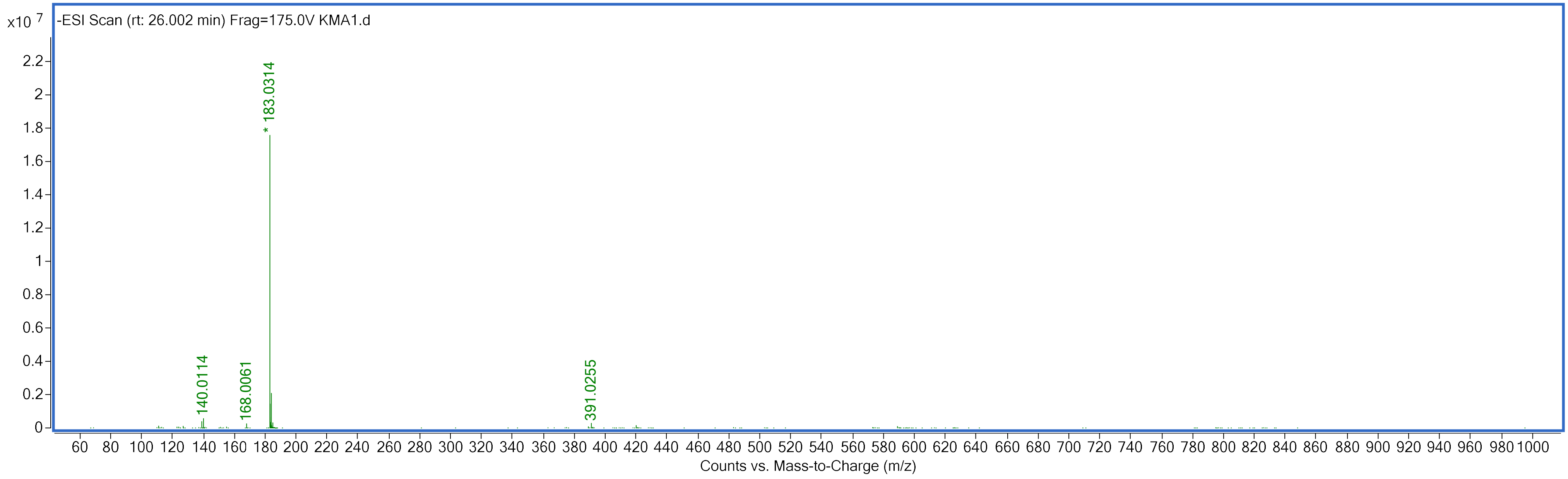

Signal 9: 27.576 min. Mangiferin ($C_{19}H_{18}O_{11}$)

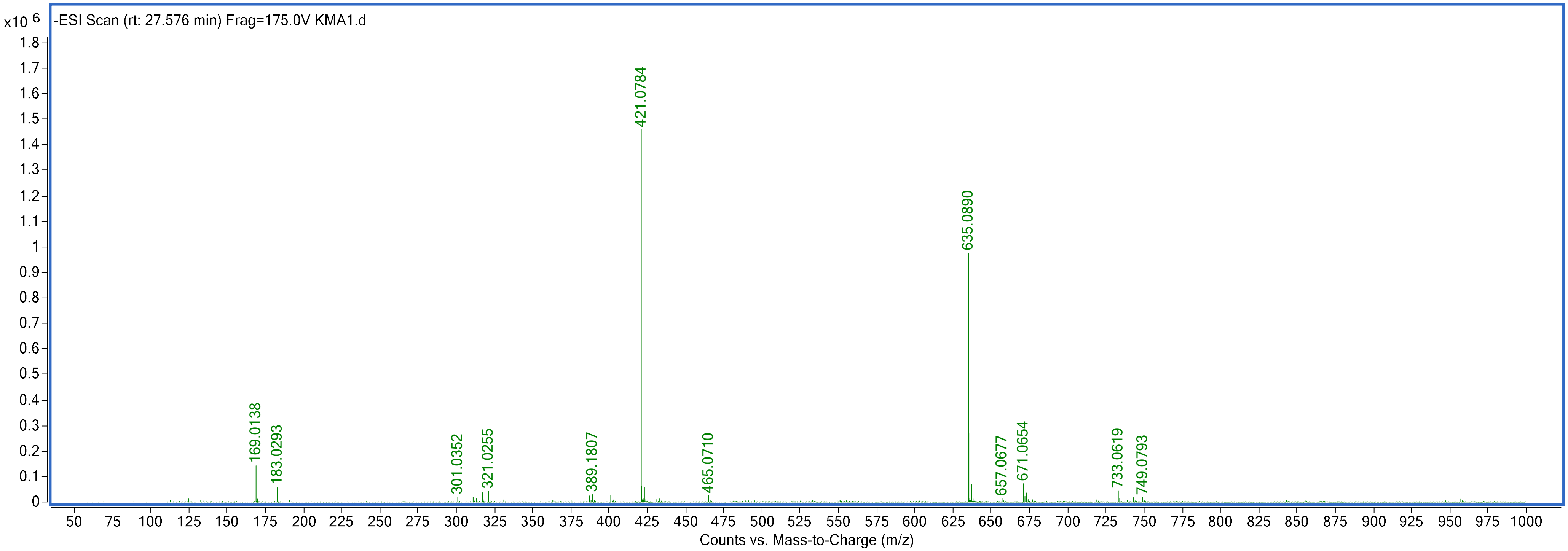

Signal 10: 28.421 min. Trigalloyl glucose Isomer I ($C_{27}H_{24}O_{18}$)

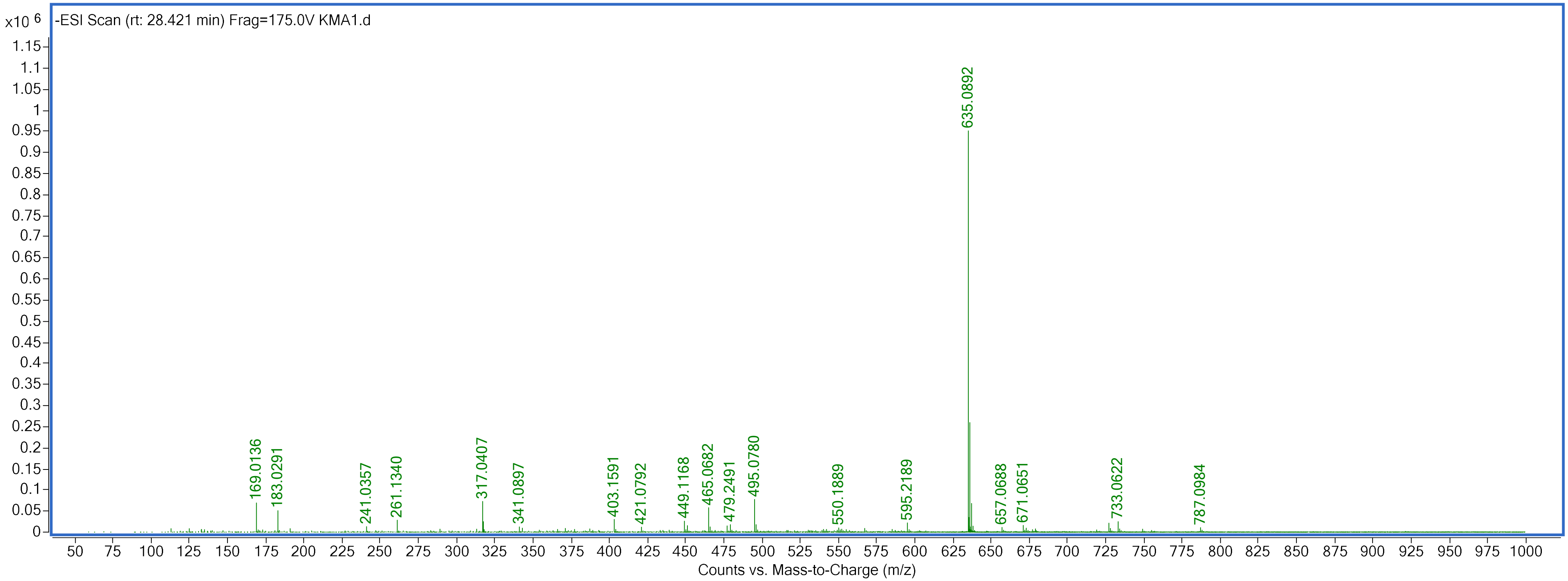

Signal 11: 28.753 min. Trigalloyl glucose Isomer II ($C_{27}H_{24}O_{18}$)

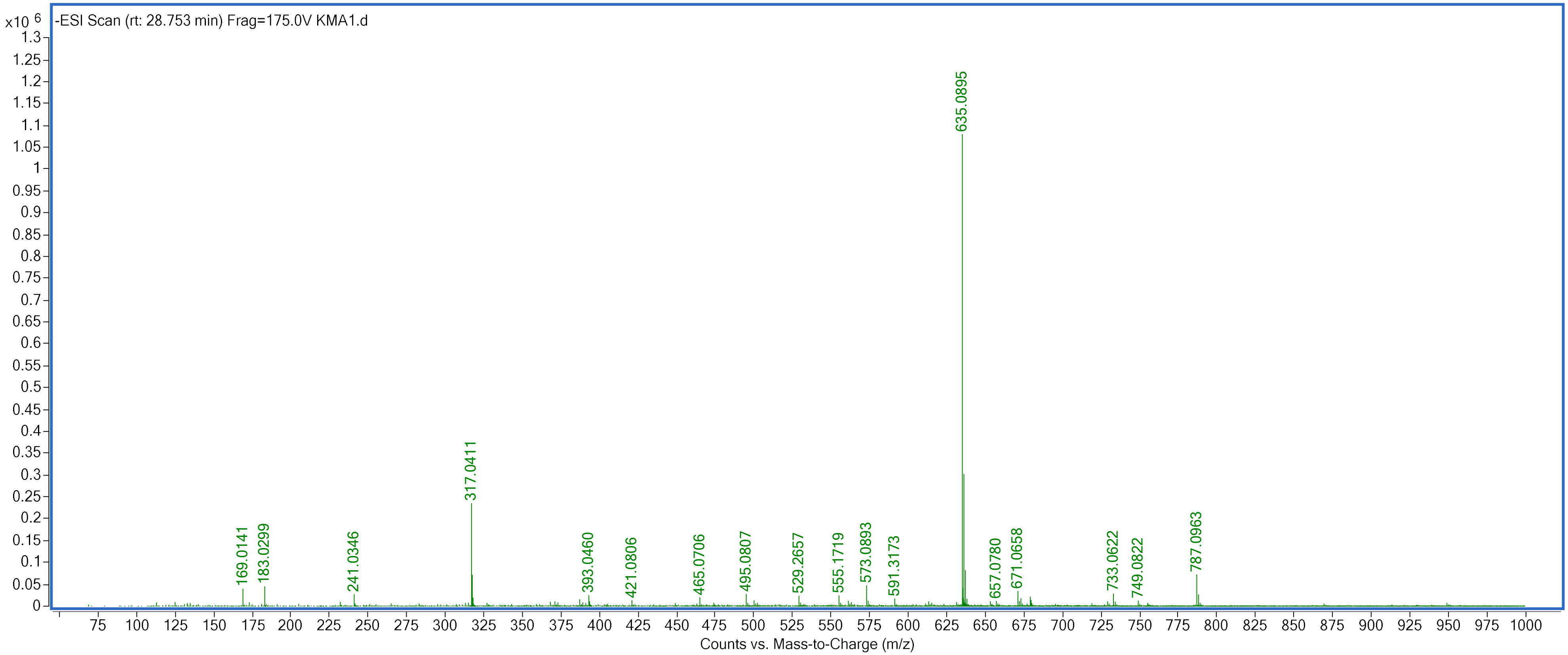

Signal 12: 29.068 min. Tetragalloyl glucose Isomer I ($C_{34}H_{28}O_{22}$)

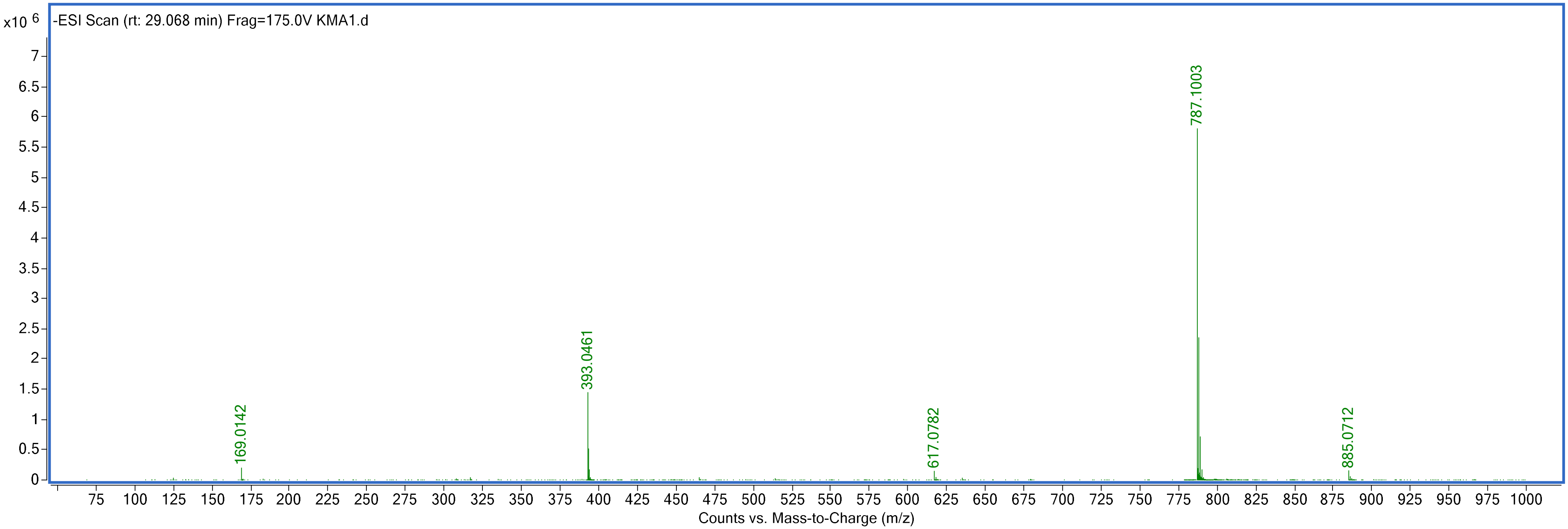

Signal 13: 29.383 min. Tetragalloyl glucose Isomer II ($C_{34}H_{28}O_{22}$)

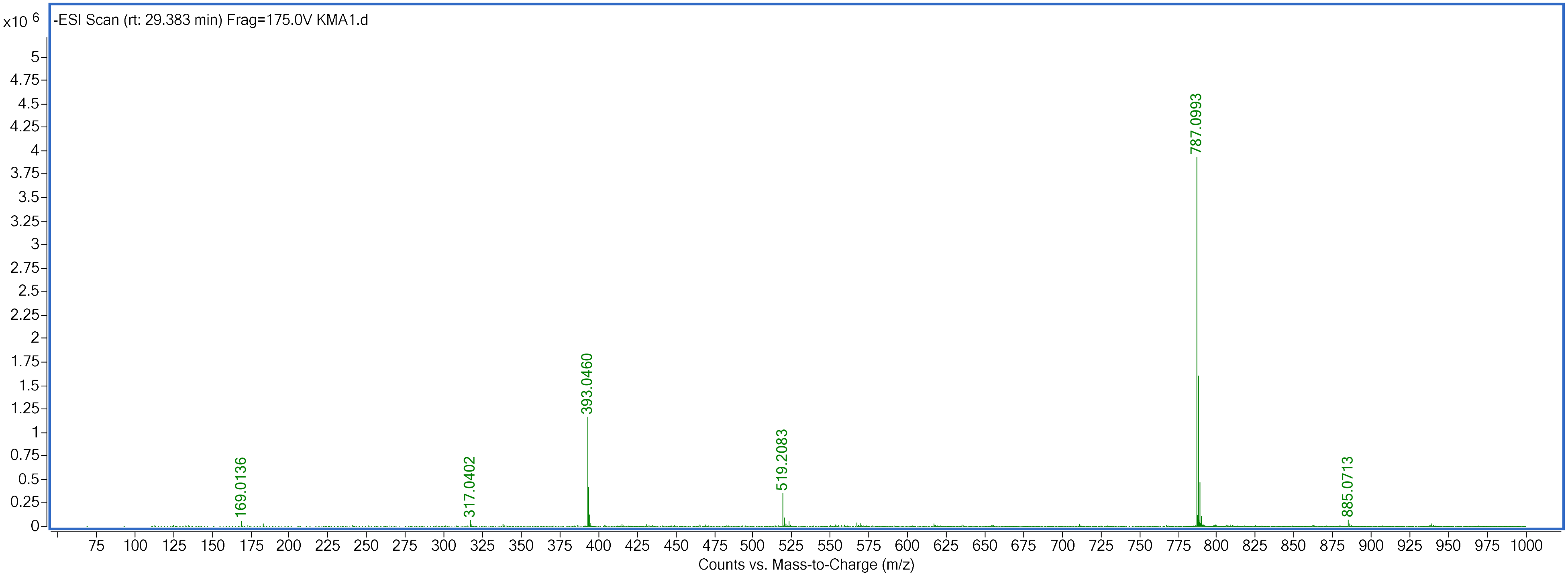

Signal 14: 29.581 min. Tetragalloyl glucose Isomer III ($C_{34}H_{28}O_{22}$)

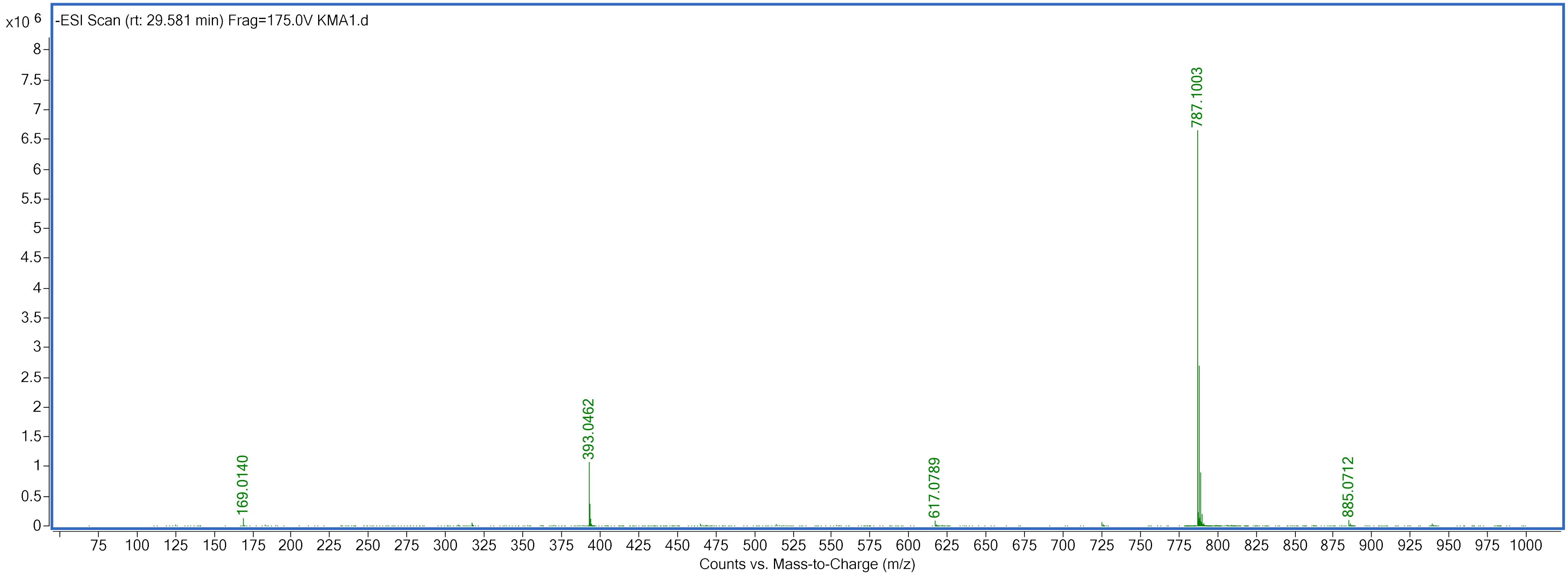

Signal 15: 29.929 min. Pentagalloyl glucose ($C_{41}H_{32}O_{26}$)

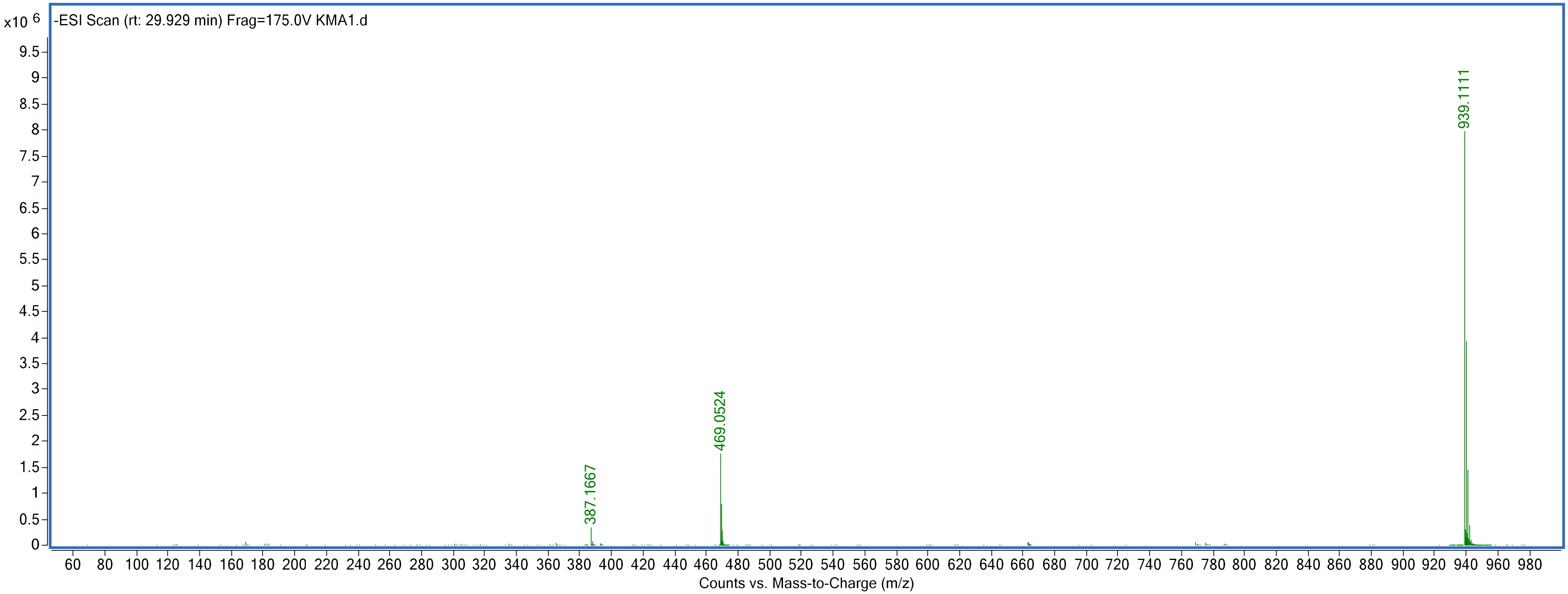

Signal 16: 30.675 min. Methyl-digallate ester ($C_{15}H_{12}O_9$)

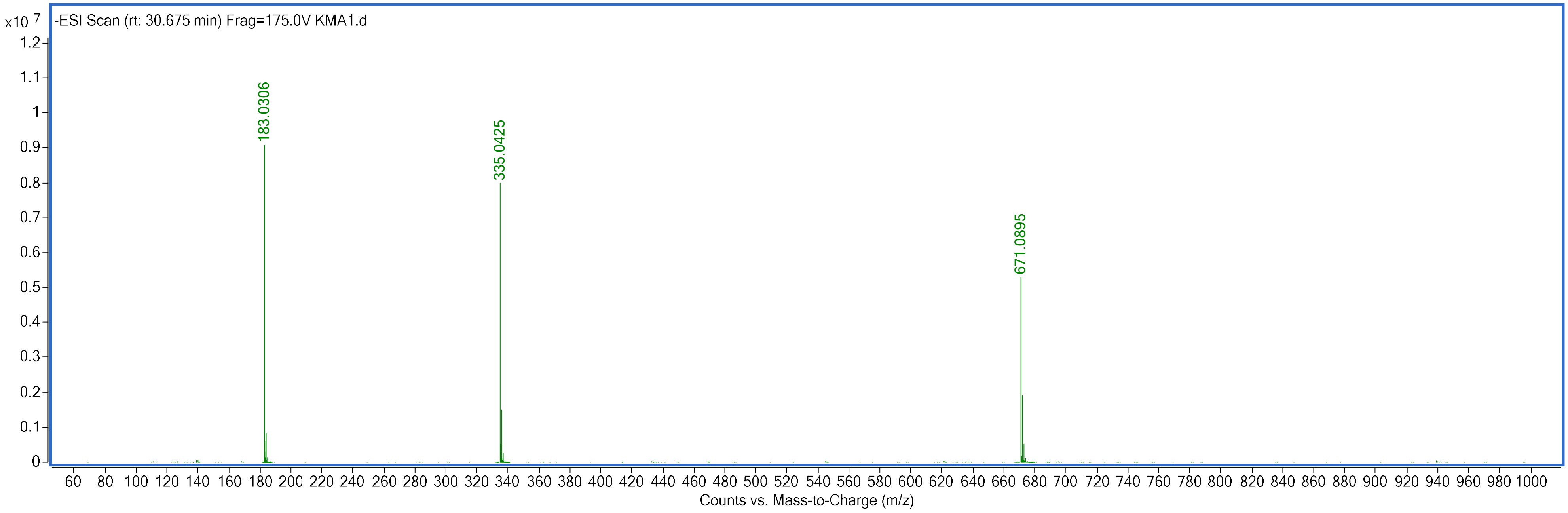

Signal 17: 31.172 min. NI ($C_{41}H_{32}O_{24}$)

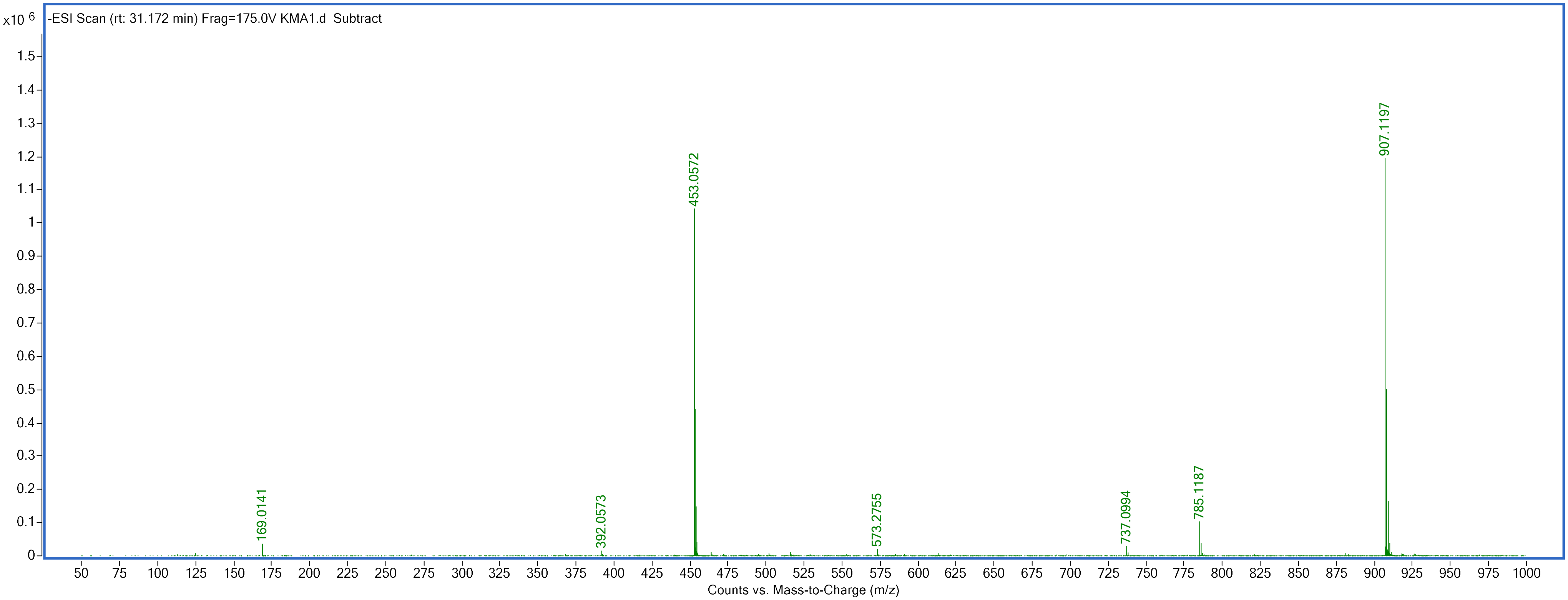

Signal 18: 33.094 min. NI

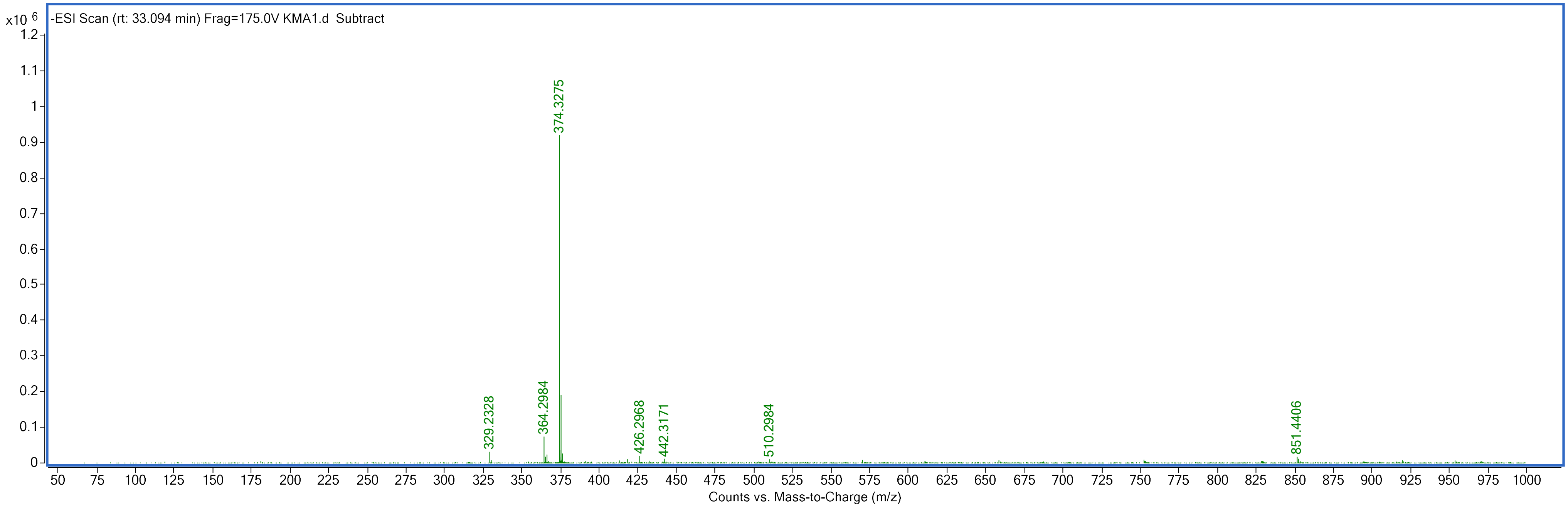

Signal 19: 33.889 min. NI

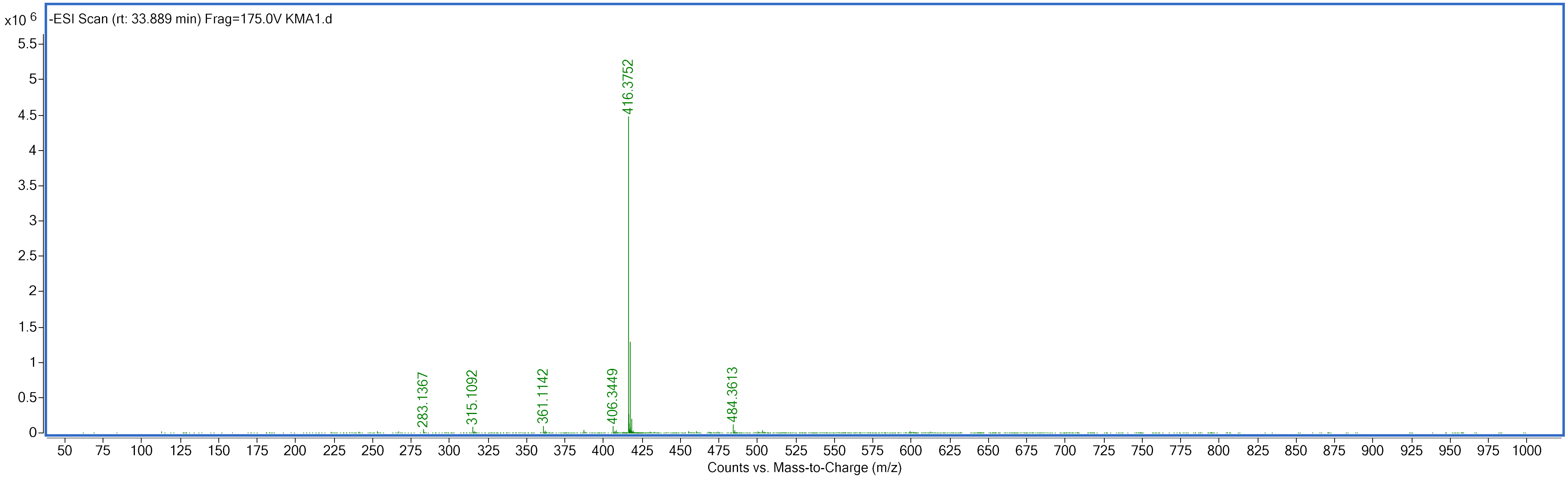

Signal 20: 46.863 min. Anacardic acid ($C_{22}H_{32}O_3$)

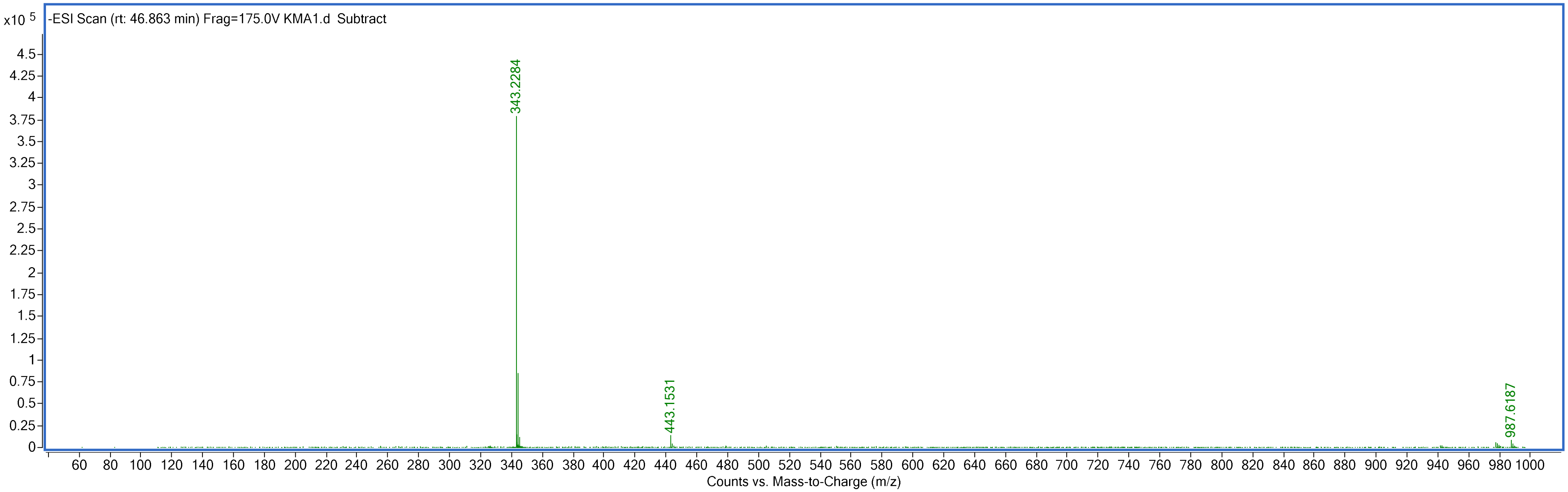

Signal 21: 48.504 min. Ginkgoic acid ($C_{22}H_{34}O_3$)

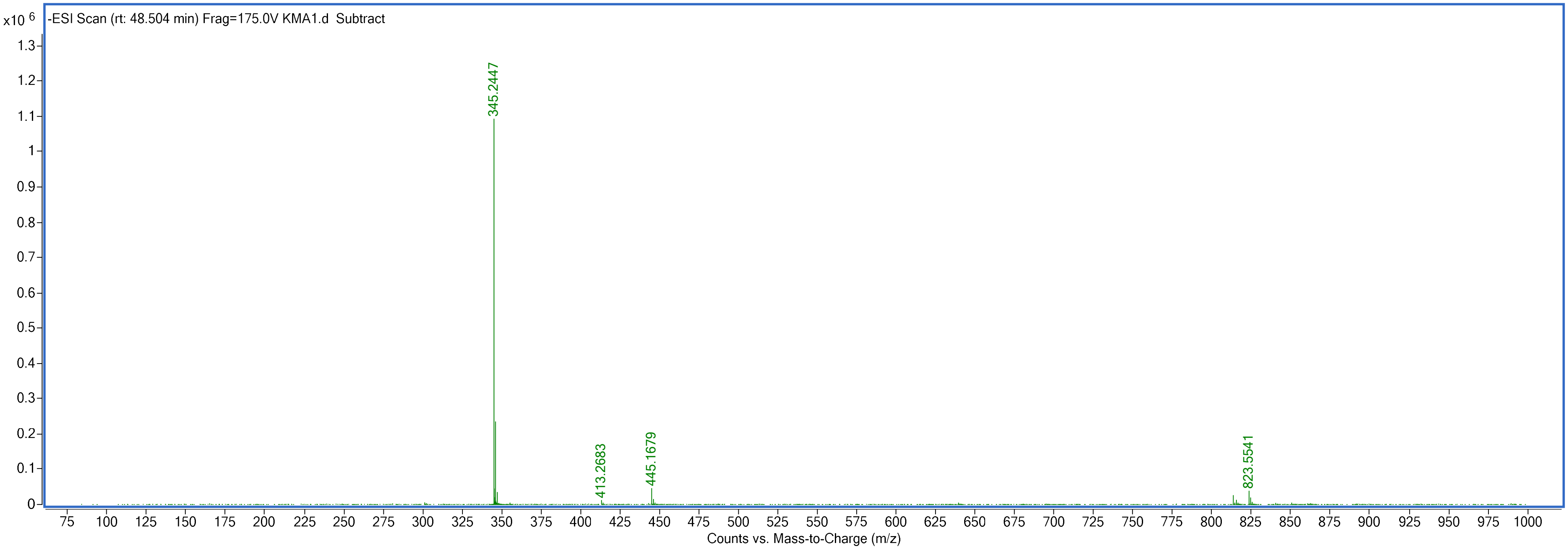

Signal 22: 49.001 min. NI($C_{24}H_{36}O_3$)

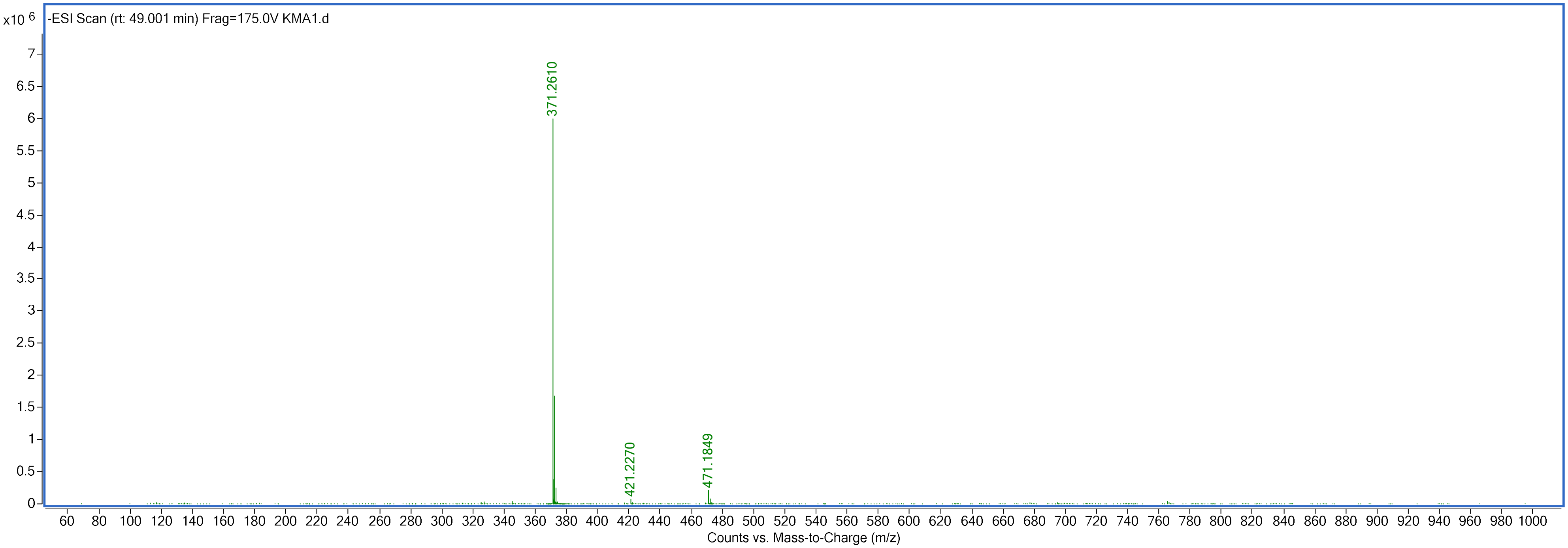

Supplement: Supplementary file 1 [file antioxidants-08-00041-s001.zip › Supplementary files/Figure S3.pdf]
