# Supplementary figures and images for: Bioactive Phenolic Compounds from the Agroindustrial Waste of Colombian Mango Cultivars ‘Sugar Mango’ and ‘Tommy Atkins’—An Alternative for Their Use and Valorization
Source: Antioxidants (Basel). 2019 Feb 15;8(2):41. doi: 10.3390/antiox8020041 (PMC6406469; doi:10.3390/antiox8020041)

Figure S2. Chromatographic profile of SKS extract obtained by HPLC-ESI-MS(Q-TOF)

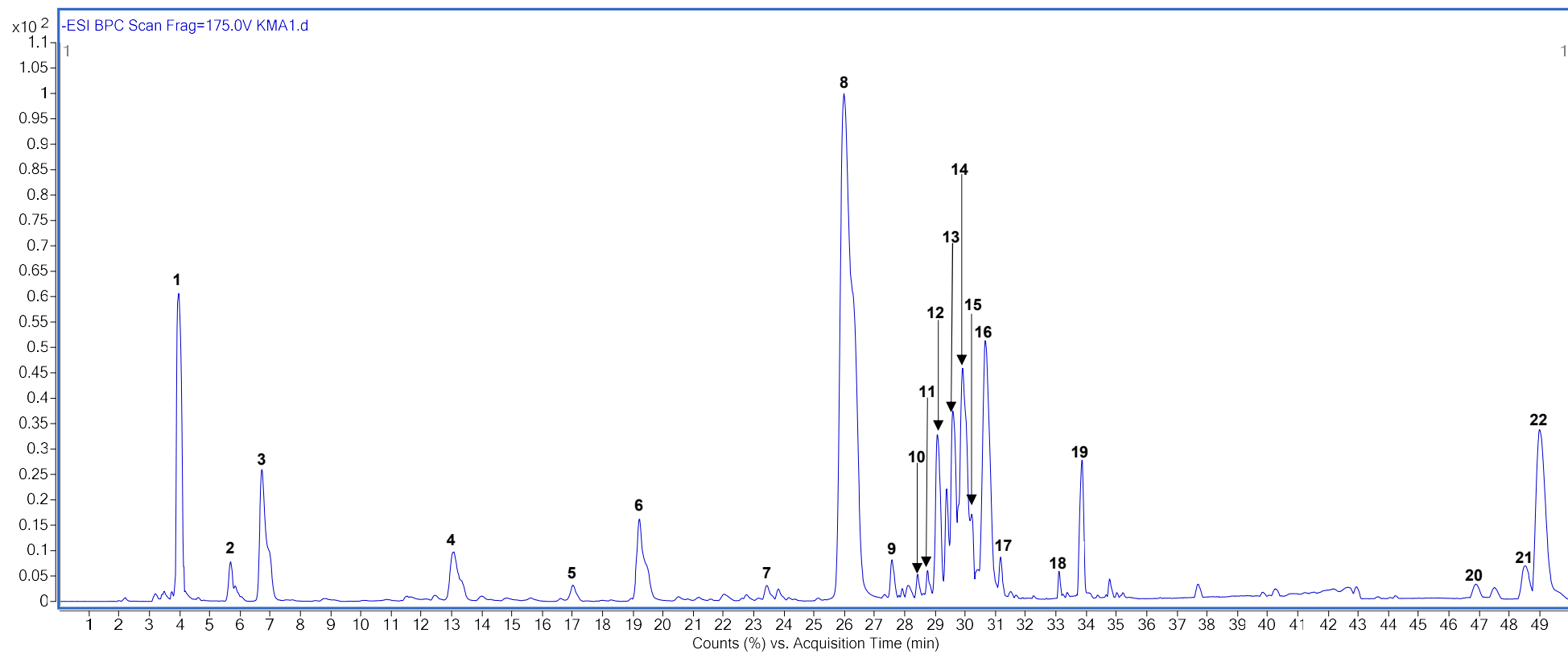

Supplement: Supplementary file 1 [file antioxidants-08-00041-s001.zip › Supplementary files/Figure S2.pdf]
